# A new member of a class of rod-like Mn_12_ single molecule magnets using 2-(pyridine-2-ly)propan-2-ol†

**DOI:** 10.1039/c9ra06280g

**Published:** 2019-11-20

**Authors:** En-Che Yang, Shi-Yi Huang, Wolfgang Wernsdorfer, Ling-Xuan Hong, Marko Damjanovic, Lukas Niekamp, Gene-Hsiang Lee

**Affiliations:** Department of Chemistry, Fu Jen Catholic University Hsinchuang, New Taipei City 24205 Taiwan Republic of China 071549@mail.fju.edu.tw +886-2-2905-3571; Institute of Nanotechnology (INT), Karlsruhe Institute of Technology (KIT) Hermann-von- Helmholtz-Platz 1, D-76344 Eggenstein-Leopoldshafen Germany; Instrumentation Centre, College of Science, National Taiwan University Taipei 10672 Taiwan Republic of China

## Abstract

This paper reports on the synthesis, structure and magnetic properties of a new type of rod like Mn_12_ metal cluster, [Mn_12_O_7_(OH)_2_(OMe)_2_(dmhmp)_4_(O_2_CPh)_11_(H_2_O)] (6) where the ligand (dmhmpH) is 2-(pyridine-2-yl)propan-2-ol. Compound (6) was obtained by reacting MnCl_2_·4H_2_O with dmhmpH in the presence of benzoic salt and Et_3_N. The resulting crystalline material is assigned to the triclinic space group *P*1̄. Although compound (6) displays ferromagnetic and antiferromagnetic competition behavior, this does not prevent the molecule from functioning as a single-molecule magnet (SMM). The SMM behavior is evidenced by observing frequency dependent out-of-phase ac signals as well as magnetization hysteresis loops at low temperatures in a micro-SQUID study. A brief comparison between all rod-like Mn_12_ materials is also given in the manuscript.

## Abstract 2

A new member of rod-like Mn_12_ molecules functions as a single molecule magnet.

## Graphical abstract

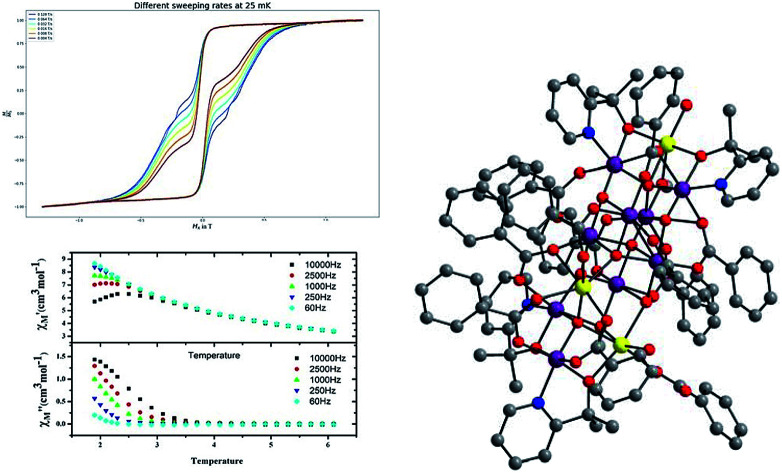

## Introduction

Because of their natural abundance and the fact that they are involved in numerous biologically related processes, 1^st^ row transition metal ions have attracted the interest of the scientific community.^1^ For example, manganese plays a key role in photo system II (PSII).^2^ Oxo-bridged manganese clusters are of great interest because of their reactivity, redox properties, ferroelectricity as well as their magnetochemistry.^3^ In applications in magnetochemistry, the most important branch is single-molecule magnets (SMMs).^4^ Single molecule magnets (SMMs) are metal clusters that simultaneously have large molecular spins and significant negative magneto-anisotropy. Upon combining these two factors, an energy barrier for spin flip is created. The manganese ion, especially Mn(iii), due to its high spin number *S* = 2, and the negative anisotropy parameter resulting from the elongation of Jahn–Teller distortion, make them ideal candidates for constructing SMMs.^5^ Since the discovery of the first SMMs [Mn_12_O_12_(O_2_CR)_16_(H_2_O)_*x*_] (*x* = 3 or 4) in 1993,^6^ a dynasty of SMMs has arisen, not only concerning their magnetism but also metal-oxo chemistry.

To control the structure for SMMs, one of the most important factors is the geometry of the ligand. The most widely used ligand for the construction of SMMs, except for the carboxylic acid, is hydroxymethylpyridine (hmpH).^7^ By using hmpH and hydroxyethylpyridine (hepH), Christou and co-workers successfully prepared a new type of “rod-like” Mn_12_ :[Mn_12_O_8_Cl_4_(O_2_CPh)_8_(hmp)_6_] (1), [Mn_12_O_8_Cl_4_(O_2_CPh)_8_(hep)_6_] (2) and [Mn_12_O_8_Br_4_(O_2_CPh)_8_(hep)_6_] (3) in 2002.^8^ In 2009, they changed the ligands from hmpH to dmhmpH dimethylhydroxy-methylpyridine. Afterward, [Mn_7_O_3_(OH)_3_(O_2_CtBu)_7_(dmhmp)_4_] (4) and [Mn_12_O_7_(OH)(MeO)_2_(O_2_CPh)_12_ (dmhmp)_4_(H_2_O)] (5) were prepared by two very different reaction procedures.^9^ At first glance, (4) and (5) appear to have very different structures, which can be attributed to very different reaction conditions used. In the process of modifying the reaction conditions for preparing Mn_7_ (4), but switching the carboxylate ligand from pivalic to benzoic acid and the use of a more MeOH rich solution system, quite surprisingly, we obtained a new Mn_12_ complex. This compound has the formula [Mn_12_O_7_(OH)_2_(OMe)_2_(dmhmp)_4_(O_2_CPh)_11_(H_2_O)] (6) which is different from compound (5). In this paper, we report on a detailed study of this accidentally prepared compound and also compare it with the other rod-like Mn_12_ complexes.

## Results and discussion

### Synthesis and structures

Compound 6 was produced by treating dmhmpH with MnCl_2_·4H_2_O, NaO_2_CPh, in the presence of Et_3_N in a solution of MeCN/MeOH (1/29 mL). It is important to use a ratio of MeCN/MeOH that is rich in MeOH, otherwise desired compound 6 is not produced in high yields.

**Fig. 1:**
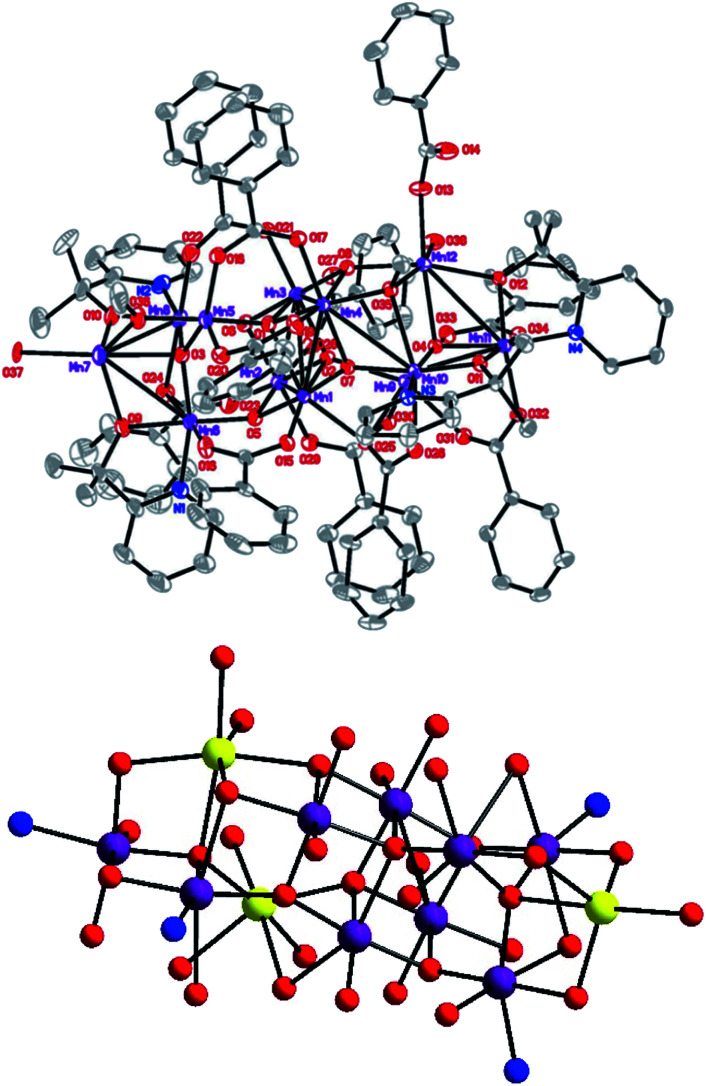
(Top) ORTEP plot of complex 6 at the 30% probability level (Mn, purple; O, red; N, blue; C, gray). (Bottom) Framework of compound 6 (Mn^II^ yellow, Mn^III^ purple, O red, N blue).

The ORTEP plot for compound 6 is shown in Fig. 1. The metal–ligand bond lengths are listed in Table 1. On calculating the charge by their structural data, and combining these with the BVS result,^10^ unlike compound 1–3 where the cation has a charge of +34, compound 6, with a charge +33, is more similar to compound 5. Although, unlike compound 1–3 which possess inversion symmetry, all of these complexes have similar structural characteristics with a central [Mn_4_O_6_] unit of uncompleted dicubane connected with two [Mn_4_O] tetrahedral units on both two ends of the rod. Compounds 6 and 5 have no intramolecular symmetry. All of compounds 1–3 contain two Mn(ii) (Mn(6) and Mn(6′) in ref. 8) ions located on the two tips on the end of the rod. In compounds 5 and 6, there are three Mn(ii) ions, one of which being located on the tip of one end, the other two being located on a lateral of the tetrahedral on the other end but does not occupy the tip position. In a comparison of compounds 5 and 6, their ligands are obviously different. Compound 5 has twelve PhCOO^−^ units, while compound 6 has eleven PhCOO^−^ units. A detailed study of the structure of 6 revealed that the whole metal-oxo core is enclosed in a package formed from four dmhmp^−^ ligands and eleven PhCOO^−^ units, where each dmhmp^−^ binds a Mn(iii) ion. The PhCOO^−^ units have three different binding modes: one monodentate η^1^ type coordinated with one manganese ion, another type μ_2_ that bridges two metal ions in a η^1^:η^1^ fashion. The final μ_3_ bridges three metal ions in a η^1^:η^2^ manner. (For detailed information on the binding modes, see Fig. S1.†)

**Table 1:** Metal–Ligand bond lengths of compound 6

Mn(1)–O(5)	1.877(2)	Mn(5)–O(1)	1.850(2)	**Mn(9)–O(30)**	**2.138(2)**
Mn(1)–O(7)	1.899(2)	Mn(5)–O(36)	1.894(2)	**Mn(9)-O(4)**	**2.1532(19)**
Mn(1)–O(15)	1.968(2)	Mn(5)–O(3)	1.930(2)	**Mn(9)-O(33)**	**2.157(2)**
Mn(1)–O(2)	1.968(2)	Mn(5)–O(18)	2.015(2)	**Mn(9)-O(31)**	**2.195(2)**
Mn(1)–O(25)	2.212(2)	Mn(5)–O(20)	2.101(3)	**Mn(9)-O(2)**	**2.279(2)**
Mn(1)–O(1)	2.271(2)	Mn(6)–O(5)	1.849(2)	**Mn(9)-O(25)**	**2.321(2)**
Mn(2)–O(6)	1.929(2)	Mn(6)–O(9)	1.896(2)	Mn(10)–O(7)	1.873(2)
Mn(2)–O(29)	1.953(2)	Mn(6)–O(3)	1.900(2)	Mn(10)–O(11)	1.877(2)
Mn(2)–O(23)	2.004(3)	Mn(6)–N(1)	2.020(3)	Mn(10)–O(4)	1.906(2)
Mn(2)–O(2)	2.007(2)	Mn(6)–O(16)	2.117(3)	Mn(10)–N(3)	2.039(3)
Mn(2)–O(28)	2.069(3)	Mn(6)–O(24)	2.479(3)	Mn(10)–O(26)	2.214(2)
Mn(2)–O(5)	2.083(2)	**Mn(7)-O(36)**	**2.088(3)**	Mn(10)–O(35)	2.377(2)
Mn(3)–O(6)	1.882(2)	**Mn(7)-O(9)**	**2.142(3)**	Mn(11)–O(12)	1.889(2)
Mn(3)–O(8)	1.923(2)	**Mn(7)-O(10)**	**2.150(3)**	Mn(11)–O(4)	1.894(2)
Mn(3)–O(2)	1.932(2)	**Mn(7)-O(37′′)**	**2.190(8)**	Mn(11)–O(32)	1.942(2)
Mn(3)–O(21)	1.972(2)	**Mn(7)-O(37′)**	**2.253(9)**	Mn(11)–N(4)	2.045(3)
Mn(3)–O(27)	2.186(3)	**Mn(7)-O(3)**	**2.301(2)**	Mn(11)–O(34)	2.120(2)
Mn(3)–O(1)	2.329(2)	**Mn(7)-O(37)**	**2.341(4)**	Mn(11)–O(11)	2.277(2)
Mn(4)–O(7)	1.880(2)	Mn(8)–O(6)	1.874(2)	**Mn(12)-O(13)**	**2.132(2)**
Mn(4)–O(1)	1.884(2)	Mn(8)–O(10)	1.885(3)	**Mn(12)-O(12)**	**2.134(2)**
Mn(4)–O(35)	1.940(2)	Mn(8)–O(3)	1.913(3)	**Mn(12)-O(8)**	**2.159(2)**
Mn(4)–O(17)	1.970(2)	Mn(8)–N(2)	2.018(3)	**Mn(12)-O(38)**	**2.226(3)**
Mn(4)–O(19)	2.158(2)	Mn(8)–O(22)	2.187(3)	**Mn(12)-O(4)**	**2.269(2)**
Mn(4)–O(8)	2.303(2)	Mn(8)–O(24)	2.301(3)	**Mn(12)-O(35)**	**2.289(2)**

### Magnetic susceptibility study of compound 6

**Fig. 2:**
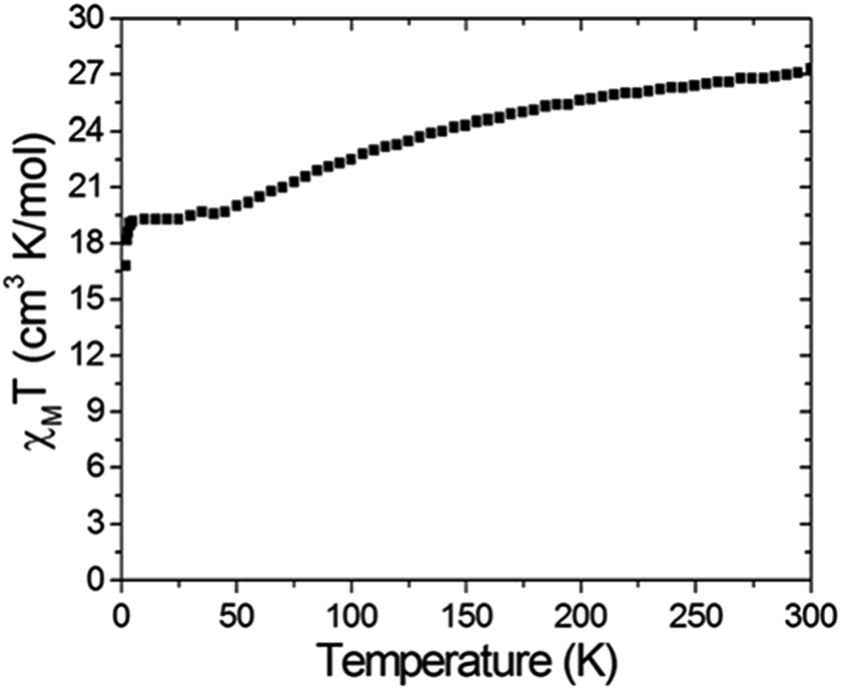
A *χ*_M_*T versus T* plot of complex 6 measured under a 1 kG magnetic field in the temperature range 2–300 K.

Variable temperature dc magnetic susceptibility measurements were carried on a polycrystalline sample of compound 6 restrained by parafilm under a 1000 G magnetic field in the temperature range of 2.0–300 K. The *χ*_M_*T vs. T* data are shown in Fig. 2. It can be seen that the *χ*_M_*T* value is 27.3 cm^3^ K mol^−1^ at 300 K, which is significantly smaller than the spin-only value: 40.13 cm^3^ K mol^−1^ for nine uncoupled Mn(iii) and three Mn(ii) ions. This phenomenon indicates that antiferromagnetic coupling plays an important role at room temperature. The *χ*_M_*T* values then steadily decrease to 20.0 cm^3^ K mol^−1^ as the temperature is cooled down to 50 K and then is nearly constant down to 4 K followed by a drop to 16.8 cm^3^ K mol^−1^ at 2 K. The abnormal feature of the *χ*_M_*T* values indicate a ferromagnetic and antiferromagnetic competition behavior in compound 6. Nevertheless, the *χ*_M_*T* value down to 2.0 K roughly gives a *S* = 11/2 ground state with a *g* value of 1.95. The *g* value deviating from 2.0 can be attributed to intermolecular antiferromagnetic coupling and/or zero-field splitting.

**Fig. 3:**
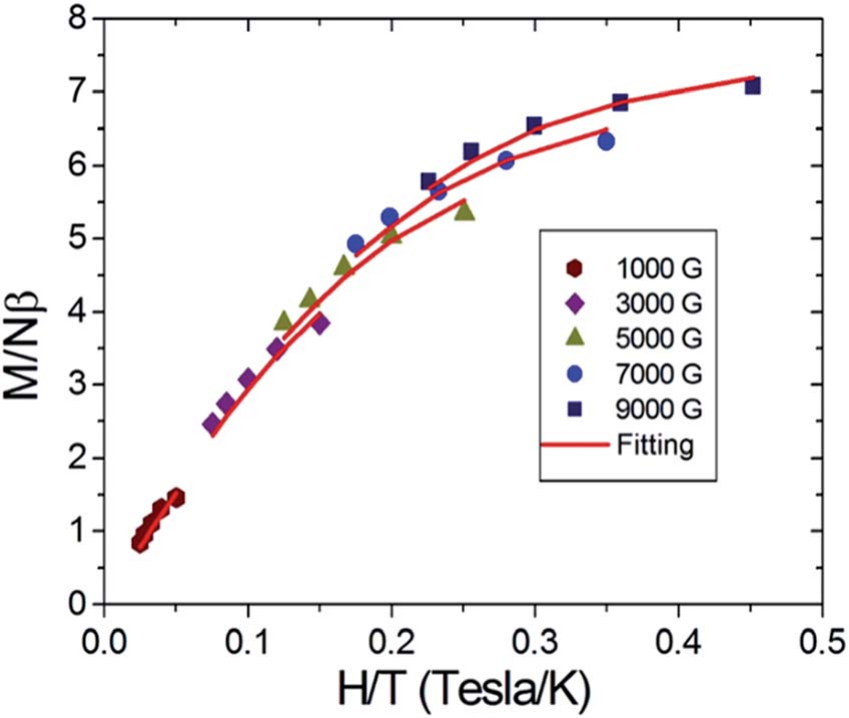
Reduced magnetization of complex 6 under magnetic field 0.1–0.9 T in the temperature range 2–4 K. Red solid lines denote the best fitting to the experimental data which give: *S* = 11/2, *g* = 2.0, and *D* = −0.34 K.

The spin number as well as the zero-field splitting parameters were then obtained by variable fields, variable temperatures reduced magnetization measurements. Based on the known information regarding compound 6, it is a ferromagnetic and antiferromagnetic competition case in which low lying high spin excited states may approach to the ground state in energy. To avoid the interference of high spin excited states and to obtain the parameter of the low spin ground state, a lower magnetic field should be applied.^11^ Therefore, in this experiment, magnetic fields in the range from 1000 G to 9000 G were applied in the temperature range of 2–4 K. The experimental results are illustrated in Fig. 3, where the solid red lines are the fittings based on eqn (1).1
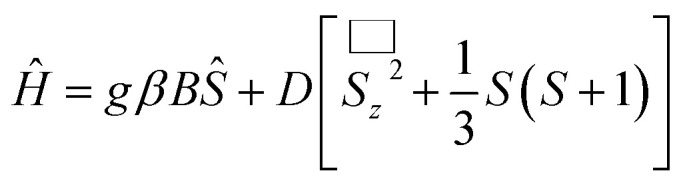


It can be seen that the eqn (1) gives a reasonably good fitting and reasonable spin parameters are obtained which gives *S* = 11/2, *g* = 2.0, *D* = −0.34 K. Based on this set of parameters, we postulate that compound 6 can function as a single-molecule magnet (SMM).

### a.c. magnetic susceptibility

**Fig. 4:**
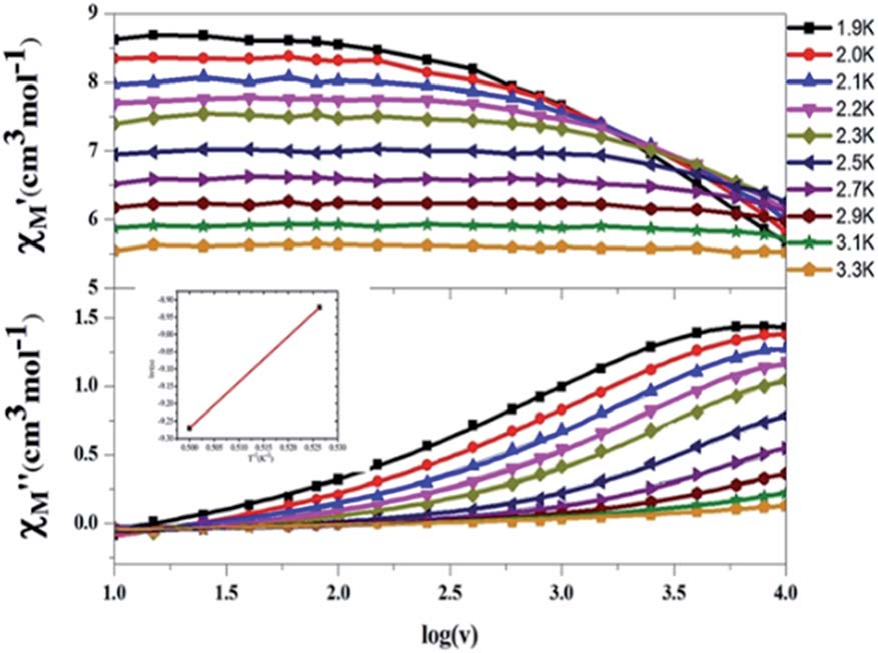
AC magnetic susceptibility data for complex 6 collected in a 3.5 G oscillating field with frequencies of 10–10 000 Hz in the temperature range 1.9–3.3 K. The in-phase signals 
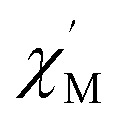
 are presented in the top, and the out-of-phase signals 
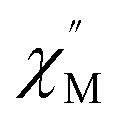
 are in the bottom. Inset: Arrhenius plot. The red solid line represents the least-squares fit.

To further confirm that compound 6 is, in fact, a SMM, a.c. magnetic susceptibility measurements were carried. In the first attempt, we applied typical measurements on the sample which applies to an a.c. magnetic field 3.5 G in the temperature range 1.9–9.0 K with the frequencies below 1000 Hz. Although frequency-dependent out-of-phase signals were seen, none of the signal showed peak feature above 1.9 K. We therefore examined a wider scope of oscillating frequencies from 10 to 10 000 Hz in the temperature range 1.9–6.1 K. Fig. 4 presents the results of temperature dependent and frequency dependent (log *ω*) scans, which show two peaks above 1.9 K. The energy barrier for spin flipping was estimated by the Arrhenius formula:^12^

2 ln(*τ*) = ln(*τ*_0_) + *U*_eff_/*kT*

which is shown in the inset of Fig. 4 to give a value of *U*_eff_ = 9.2 K. This result is somewhat lower than the theoretical value *D*(*S*^2^ − 0.25) = 10.2 K. This implies that somewhat quantum tunnelling of magnetization (QTM)^13^ occurs at this moment. Armed with this data, it can be concluded that compound 6 is a SMM.

**Fig. 5:**
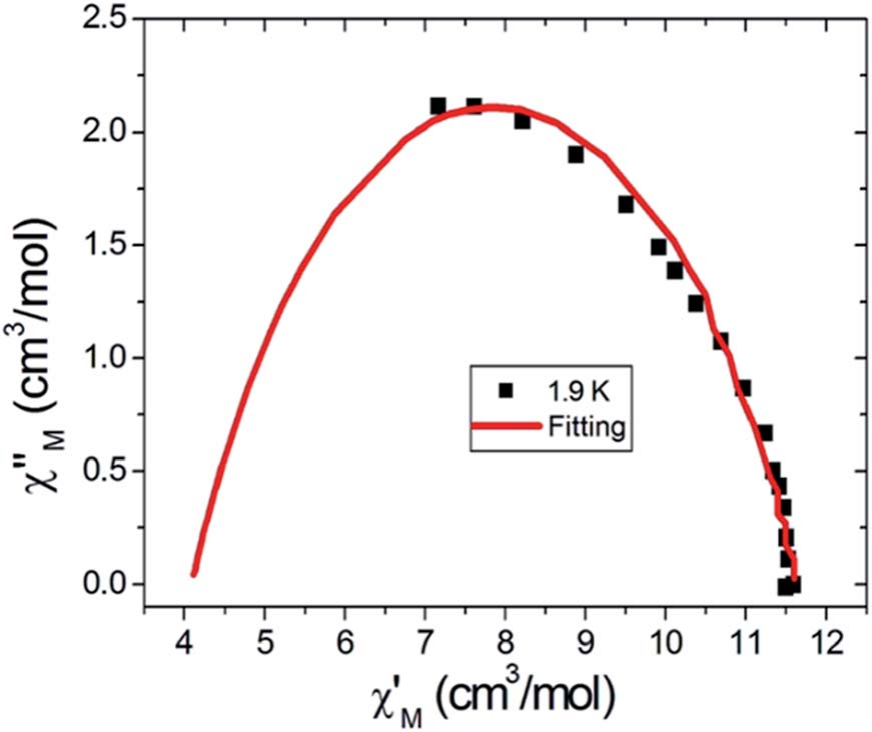
Cole–Cole plot at 1.9 K for complex 6. The red solid line is the least-square fit of the data to eqn (3) and (4).

Further detail a.c. magnetic susceptibility examinations regarding magnetization relaxation process was analysed by the Debye model of Argand's plot (
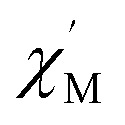
*vs.*
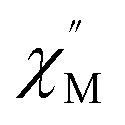
 plot) which is also known as Cole–Cole plot.^14^ The Debye model is described as eqn (3) and (4):3
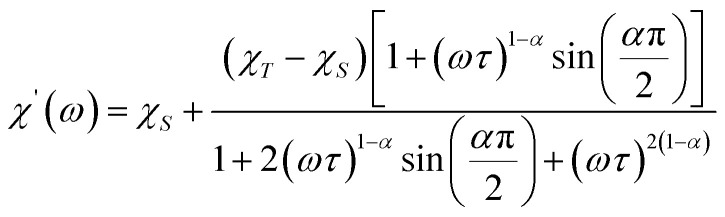
4
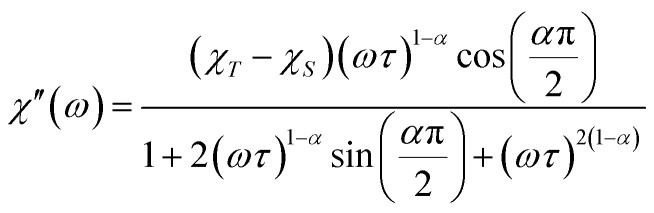
where 
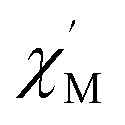
 and 
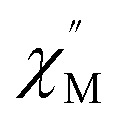
 are the in-phase and out-of-phase a.c. magnetic susceptibilities. Both are the functions of scanning frequencies *ω*. The parameters *χ*_S_ represents the adiabatic susceptibility, *χ*_T_ the isothermal susceptibility and *τ* the magnetization relaxation time. The value for parameter *α* varies in the range 0–1 and provide an estimation of the distribution of relaxations. An Argand's plot for compound 6 at 1.9 K is shown in Fig. 5. The parameters in eqn (3) and (4) obtained from the best fitting are given as *χ*_T_ = 11.64 cm^3^ mol^−1^, *χ*_S_ = 4.09 cm^3^ mol^−1^, *α* = 0.35, and *τ* = 2.03 × 10^−3^ s. From this result, we can conclude a distribution of single relaxation for compound 6. This was also observed in many other manganese based single molecule magnets (SMMs).

### Magnetization hysteresis studies

**Fig. 6:**
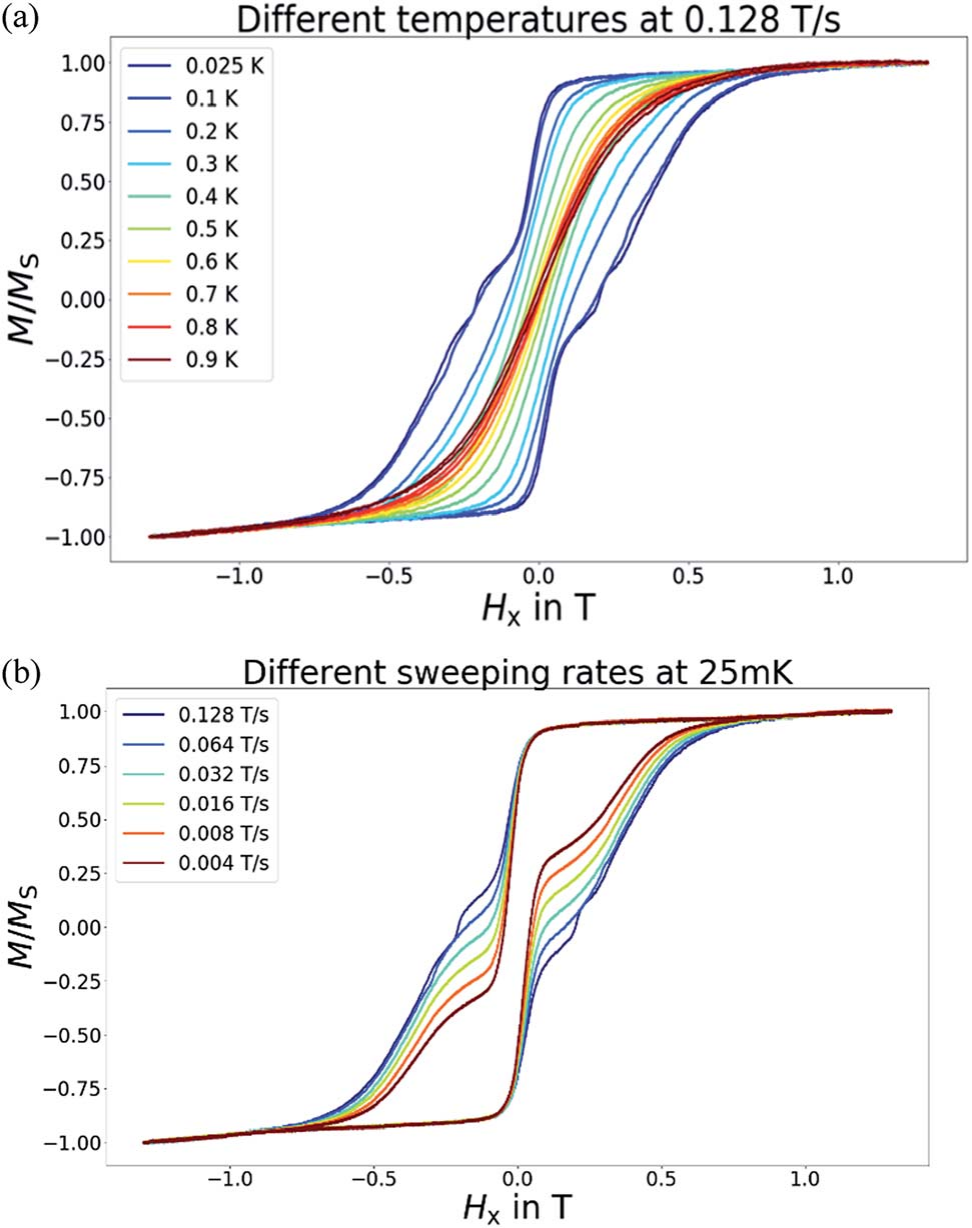
Magnetization hysteresis measurements for compound 6. (a) Experiments were performed at a scanning rate of 0.128 T s^−1^ in the temperature range of 0.025–0.9 K. (b) Hysteresis loops were collected at a constant temperature (25 mK) with scanning rates in the range 0.004–0.128 T s^−1^.

Magnetization hysteresis measurements were then carried on a single crystal of 6 in a micro-SQUID array with the magnetic field aligned with the easy axis of the crystal. Fig. 6(a) presents the hysteresis loops observed in the temperature range 0.025–0.9 K. Obviously, the hysteresis loops can be seen at temperatures below 0.9 K providing an indication of magnet type behavior. Upon cooling, the magnitude for the coercivity of each loop increases until the lowest reachable temperature 25 mK, which reflects the fact that slower magnetization relaxation occurs at lower temperatures. Since the magnetization relaxation process appears to be dominated by quantum tunnelling of the magnetization (QTM) at 25 mK, to explore the nature of the QTM, we examined the hysteresis loops when the temperature is maintained at 25 mK but varying the scan rate in the range of 0.004 T s^−1^ to 0.128 T s^−1^. Several points are worth mentioning in Fig. 6(b). First, all the loops show a giant transition at zero magnetic field that can be attributed to the QTM process between the ground spin states. The fact that this occurs at zero field indicates that there is no significant intermolecular interaction to shift the degeneracy of the ground states of spin multiplicity. In another words, no significant intermolecular exchange bias^15^ occurs in this crystal system. Secondly, the step occurring at zero field increase in size when the scanning rate is slowed. This seems to be the result of a longer time interval the molecule system stays in the QTM window the larger is the QTM amplitude and therefore bigger transition steps are seen. Compared with most typical manganese SMMs [Mn_12_O_12_(O_2_CR)_16_(H_2_O)_3∼4_],^16^ the step feature for compound 6 is significantly larger, we therefore conclude that compound 6 belongs to the category of SMMs with a fast QTM. Of course, compound 6 possesses the molecular spin *S* = 11/2, a half integer spin that might hinder the QTM process. Nevertheless, a combination of a hyperfine interaction of ^55^Mn (I = 5/2, 100%) as well as the lack of symmetry axis of C_3_ and/or above which introduces a transverse field, can still trigger a fast QTM in compound 6.^17^

### Comparisons with other rod-like Mn_12_ complexes

According to the literature, four rod-like Mn_12_ complexes have been reported in which two are SMMs. Combining with the compound 6, all five compounds were synthesized using a hydroxyl-alkyl-pyridine type of ligand. Changing the alkyl group slightly causes significant differences in the magnetic properties of these complexes. In compound 1, pure hmp ligands with alkyl = methylene leads to a SMM with *S* = 7(or 6), all of the ac frequencies above 10 Hz have their out-of-phase signals above 1.9 K. Compounds 2 and 3, both contain hep ligands with alkyl = ethyl groups. No SMMs behavior was observed in this series compounds which have molecular spin *S* = 0. Compound 5 and the current work 6 contain dmhmp with alkyl = isopropyl groups. Although both exhibit SMM behavior, their spin parameters are somewhat different. Compound 5 has molecular spin *S* = 13/2 and all of its ac out-of-phase signals show only a tail above 1.9 K. However, promising magnetization hysteresis loops have been reported at temperatures below 0.7 K. Thus, compound 5 is a SMM. The current compound 6 also contains alkyl = isopropyl. However, its peripheral ligands are arranged slightly different from those for compound 5 with two coordinated hydroxide ion and a dangling benzoic acid ligand. In this compound, frequency-dependent out-of-phase signals are seen, and two peaks above 1.9 K are seen with frequencies in the interval 1000–10 000 Hz. Solid magnetization hysteresis loops are seen below 0.9 K. The obtained molecular spin for compound 6 was *S* = 11/2. Hysteresis measurements indicates that this molecular system is a fast QTM of SMM. Details regarding the comparison of these five compounds are given in Table 2 with their plots in the ESI (Fig. S2†).

**Table 2:** Comparisons of all rod-like Mn_12_ complexes^a^

	Complex	*S*	*D* (cm^−1^)	*U*_eff_ (K)
(1)	[Mn^II^_2_Mn^III^_10_O_8_Cl_4_(O_2_CPh)_8_(hmp)_6_]	6(7)	−0.40	29.1
(2)	[Mn^II^_2_Mn^III^_10_O_8_Cl_4_(O_2_CPh)_8_(hep)_6_]	0	—	—
(3)	[Mn^II^_2_Mn^III^_10_O_8_Br_4_(O_2_CPh)_8_(hep)_6_]	0	—	—
(5)	[Mn^II^_3_Mn^III^_9_O_7_(OH)(OMe)_2_(O_2_CPh)_12_(dmhmp)_4_(H_2_O)]	13/2	−0.18	11.5
(6)	[Mn^II^_3_Mn^III^_9_O_7_(OH)_2_(OMe)_2_(O_2_CPh)_11_(dmhmp)_4_(H_2_O)]	11/2	−0.24	9.2

a hmpH 2-(hydroxymethyl)pyridine – hepH 2-(hydroxyethyl)pyridine – dmhmpH 2-(pyridine-2-yl)propan-2-ol.

In conclusion, the Mn_12_ rod-like complex reported in this study exhibits competing ferromagnetic and antiferromagnetic exchange interactions. As expected because Mn(ii) ions are present in its structure these interactions are very weak leading to the existence of several low-lying excited states, as was also evidenced from variable field-variable temperature magnetization studies. Minor structural changes in the structure of such complexes can lead to small changes of the exchange interactions between the Mn ions, thus leading to a different spin ground state value. This explains why the *S* values of compounds 5 and 6 are different.

## Experimental

All chemicals used in this study were of commercial grade and were used without further purification. The ligand dmhmpH was synthesized according to a literature report.^18^

### [Mn_12_(dmhmp)_4_(PhCO_2_)_11_(CH_3_O)_2_(H_2_O)(OH)_2_O_7_] (6)

In a 100 mL beaker, the ligand dmhmpH 0.13 g (1 mmol) and triethylamine Et_3_N 0.14 mL (1 mmol) were dissolved in a mixed solvent MeCN/MeOH (1/29 mL). MnCl_2_·4H_2_O 0.2 g (1 mmol) and NaO_2_Ph 0.29 g (2 mmol) were added and the mixture was then stirred overnight. After filtration, diethylether was allowed to diffuse into the filtrate. Dark black crystals suitable for X-ray crystallographic analysis was obtained after two weeks. Selected IR data (cm^−1^): 3435(br), 2973(m), 1602(s), 1557(s), 1482(s), 1392(s), 1122(s), 1052(m), 978(s), 884(s), 840(s), 781(s). Anal. Calcd (found) for C_111_H_104_N_4_ClMn_12_O_37_: C, 47.92 (47.75); H, 3.77 (3.44); N, 2.01 (1.89).

### X-ray crystallography

X-ray analysis data for 6 was collected on a Bruker D8 Venture diffractometer with Mo Kα radiation (*λ* = 0.71073 Å). The temperature was cooled to 200(2) K. The corresponding data was collected by Bruker APEX3 program, and simplified by Bruker SAINT program. Data were corrected by the empirical absorption *SADABS* program.^19^ The *SHELXTL* program on a PC computer was used for the structure analysis. The structure was solved by direct method of the *Shelxs* program^20^ and refinement was done by the *Shelxl* program^21^ based on the full-matrix least squares on *F*^2^ values. Non-hydrogen atoms were refined anisotropically. Hydrogen atoms were fixed at the calculated positions and refined using a riding mode. Table 3 summarizes the structure refinement parameters for complex 6.

**Table 3:** Crystallographic data for complex 6^a^

Complex 6	
Formula	C_115_ H_112_ Mn_12_ N_4_ O_39_
Fw, *g* mol^−1^	2833.36
*T*/K	200(2)
Space group	*P*1̄
*a*/Å	15.8858(4)
*b*/Å	17.4589(4)
*c*/Å	27.1441(7)
*α* (°)	96.7381(6)
*β* (°)	103.1034(5)
*γ* (°)	107.7821(6)
Volume/Å^3^	6839.3(3)
*Z*′, *Z*	2
*F*(000)	2884
Density (calcd) mg m^−3^	1.376
Absorption coefficient mm^−1^	1.140
Absorption correction	Semi-empirical from equivalents
Reflns, measured	61 548
Reflns, independent	31 323 [*R*(int) = 0.0303]
Data/restraints/parameters	31 323/87/1632
Goodness-of-fit on *F*^2^	1.028
*R* indices [*I* > 2*σ*(*I*)]	*R*_1_ = 0.0474, w*R*_2_ = 0.1312
*R* indices (all data)	*R*_1_ = 0.0675, wR_2_ = 0.1458

a *R*_1_ = Σ‖*F*_0_| − |*F*_c_‖/Σ|*F*_0_|; w*R*_2_ = [Σw(*F*_0_^2^ − *F*_c_^2^)^2^/Σw(*F*_0_^2^)^2^]^1/2^; 
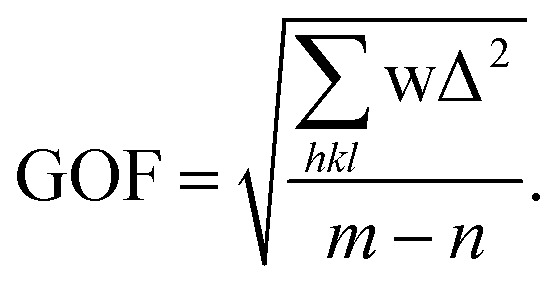

### Physical property measurements

NMR spectra were collected on a Bruker AV-300 spectrometer. Infrared spectra were obtained using KBr pellets on a PerkinElmer 1600 spectrometer in the 450–4000 cm^−1^ range. DC magnetic measurements were collected using a Quantum Design MPMS7 system. Samples were restrained with parafilm to prevent torqueing. The magnetic background caused by the gel cap and the parafilm were subtracted by blank measurements. The diamagnetism correction was estimated from Pascal's constant.^22^ Elemental analyses (C, H, N) and dc magnetic susceptibility measurements were carried by the National Taiwan University Instrument Centre, College of Science. AC magnetic susceptibility data were collected using the Quantum Design MPMS XL7 system of the National Chiao Tung University. Oriented single crystal magnetization hysteresis loops were measured by employing a micro-SQUID array that was described elsewhere.^23^ A single crystal was put onto the array and external field is oriented parallel to the crystal easy axis.

## Conclusions

Many factors in a synthetic procedure can influence the geometries of the resulting products. For example, when hmpH ligands were used, a more symmetric geometry of compound 1 was obtained. SMM type behavior was observed in this compound. While hepH was employed, two Mn_12_ clusters (2 and 3) with *S* = 0 were obtained and none were found to be SMMs. Upon switching the backbone of the hmpH ligand to isopropyl (dmhmpH), the situation becomes more sophisticated. When MnCl_2_ and tBuCO_2_^−^ are used as the reactants in a MeCN rich solution, a Mn_7_ cluster (4) was isolated which is not a SMM. When Mn(O_2_CPh)_2_ was used in a CH_2_Cl_2_ rich solution a more distorted rod like Mn_12_ (5) was isolated which was shown to be a SMM. Attempts to explore other geometries of manganese clusters by utilizing MnCl_2_ and PhCO_2_^−^ as the reactants in a MeOH rich solution, a new type Mn_12_ (6) was obtained. According to the work herein, we conclude that this compound is a SMM. Obviously, a number of factors, including peripheral ligands and solvent systems play important roles in determining the geometry of the manganese cluster and so do their magnetic properties. Although without the ability to carefully control the parameters that affect the nature of the product, this study clearly shows that approaches are available to significantly tune the magnetic properties of the metal clusters.

## Conflicts of interest

There are no conflicts to declare.

## Supplementary Material

RA-009-C9RA06280G-s001

RA-009-C9RA06280G-s002
