# Supplementary material for: A new member of a class of rod-like Mn12 single molecule magnets using 2-(pyridine-2-ly)propan-2-ol
Source: RSC Adv. 2019 Nov 20;9(65):37740–6. doi: 10.1039/c9ra06280g (PMC9075757; doi:10.1039/c9ra06280g)
Supplement: RA-009-C9RA06280G-s001 [file RA-009-C9RA06280G-s001.rtf]

Supporting Information
A New Member of a Class of Rod-Like Mn12 Single Molecule Magnets Using 2-(Pyridine-2-ly)propan-2-ol

En-Che Yang,*a Shi-Yi Huang,a Wolfgang Wernsdorfer,b Ling-Xuan Hong a, Marko Damjanovicb, Lukas Niekampb, Gene-Hsiang Lee,c

a Department of Chemistry, Fu Jen Catholic University, Hsinchuang, New Taipei City, 24205, Taiwan, Republic of China
b Institut Néel, CNRS Nanosciences Department BP 166 25 rue des Martyrs 38042 GRENOBLE Cedex 9 France
c Instrumentation Centre, College of Science, National Taiwan University, Taipei, 10672, Taiwan, Republic of China


Figure 1S. Binding modes of benzoic acid ligands to the manganese ions: (a) monodentate 1 type, (b) 2 that bridges two metal ions in a 1:1 fashion. (c) 3 bridges three metal ions in a 1:2 manner


Figure 2S. Structures of the five rod-like Mn12 complexes.


  Table 1.  Crystal data and structure refinement for ic19479_sq.
Identification code 	ic19479_sq
Empirical formula 	C115 H112 Mn12 N4 O39
Formula weight 	2833.36
Temperature 	200(2) K
Wavelength 	0.71073 Å
Crystal system 	Triclinic
Space group 	P-1
Unit cell dimensions	a = 15.8858(4) Å	a= 96.7381(6)°.
	b = 17.4589(4) Å	b= 103.1034(5)°.
	c = 27.1441(7) Å	g = 107.7821(6)°.
Volume	6839.3(3) Å3
Z	2
Density (calculated)	1.376 Mg/m3
Absorption coefficient	1.140 mm-1
F(000)	2884
Crystal size	0.328 x 0.167 x 0.103 mm3
Theta range for data collection	2.198 to 27.494°.
Index ranges	-20<=h<=20, -22<=k<=22, -35<=l<=35
Reflections collected	61548
Independent reflections	31323 [R(int) = 0.0303]
Completeness to theta = 25.242°	99.9 % 
Absorption correction	Semi-empirical from equivalents
Max. and min. transmission	0.7456 and 0.6489
Refinement method	Full-matrix least-squares on F2
Data / restraints / parameters	31323 / 87 / 1632
Goodness-of-fit on F2	1.028
Final R indices [I>2sigma(I)]	R1 = 0.0474, wR2 = 0.1312
R indices (all data)	R1 = 0.0675, wR2 = 0.1458
Extinction coefficient	n/a
Largest diff. peak and hole	1.523 and -0.716 e.Å-3

 Table 2.  Atomic coordinates  ( x 104) and equivalent  isotropic displacement parameters (Å2x 103)
for ic19479_sq.  U(eq) is defined as one third of  the trace of the orthogonalized Uij tensor.
________________________________________________________________________________ 
	x	y	z	U(eq)
________________________________________________________________________________  
Mn(1)	2952(1)	7123(1)	7908(1)	26(1)
Mn(2)	1395(1)	6430(1)	6950(1)	33(1)
Mn(3)	2416(1)	5382(1)	7214(1)	29(1)
Mn(4)	4097(1)	6065(1)	8224(1)	27(1)
Mn(5)	4713(1)	6928(1)	7305(1)	35(1)
Mn(6)	3325(1)	8061(1)	6970(1)	35(1)
Mn(7)	4754(1)	7867(1)	6343(1)	50(1)
Mn(8)	2949(1)	6369(1)	6287(1)	39(1)
Mn(9)	1116(1)	5874(1)	8181(1)	27(1)
Mn(10)	3217(1)	6448(1)	9087(1)	26(1)
Mn(11)	1678(1)	5174(1)	9300(1)	26(1)
Mn(12)	2555(1)	4524(1)	8491(1)	29(1)
O(1)	3806(1)	6445(1)	7608(1)	31(1)
O(2)	1936(1)	6079(1)	7591(1)	28(1)
O(3)	3834(2)	7236(2)	6827(1)	35(1)
O(4)	2090(1)	5574(1)	8747(1)	25(1)
O(5)	2526(2)	7487(1)	7307(1)	32(1)
O(6)	2138(2)	5910(2)	6664(1)	35(1)
O(7)	3470(1)	6710(1)	8474(1)	27(1)
O(8)	2813(1)	4937(1)	7803(1)	30(1)
O(9)	4035(2)	8643(2)	6570(1)	44(1)
O(10)	3658(2)	6811(2)	5851(1)	50(1)
O(11)	3127(1)	6068(1)	9698(1)	29(1)
O(12)	2192(1)	4347(1)	9190(1)	30(1)
O(13)	3128(2)	3579(2)	8393(1)	48(1)
O(15)	3818(2)	8217(1)	8280(1)	34(1)
O(14)	1932(3)	2454(3)	8016(2)	67(1)
O(14')	3275(6)	3562(5)	7667(4)	67(1)
O(16)	4346(2)	8759(2)	7657(1)	48(1)
O(17)	4780(2)	5394(2)	7986(1)	37(1)
O(18)	5430(2)	6177(2)	7480(1)	37(1)
O(19)	5454(2)	6963(2)	8570(1)	38(1)
O(20)	5613(2)	7755(2)	7980(1)	40(1)
O(21)	2970(2)	4699(2)	6857(1)	41(1)
O(22)	3809(2)	5628(2)	6504(1)	49(1)
O(23)	885(2)	6750(2)	6292(1)	53(1)
O(24)	2223(2)	7306(2)	6123(1)	46(1)
O(25)	1966(1)	7264(1)	8341(1)	29(1)
O(26)	2675(2)	7467(1)	9181(1)	32(1)
O(27)	1038(2)	4453(2)	6955(1)	47(1)
O(28)	313(2)	5323(2)	6708(1)	50(1)
O(29)	653(2)	6995(2)	7216(1)	39(1)
O(30)	32(2)	6161(2)	7702(1)	37(1)
O(31)	614(2)	6334(1)	8804(1)	33(1)
O(32)	1258(2)	6040(1)	9552(1)	31(1)
O(33)	157(2)	4640(1)	8042(1)	37(1)
O(34)	314(2)	4466(1)	8860(1)	35(1)
O(35)	3956(1)	5550(1)	8810(1)	29(1)
O(36)	5493(2)	7426(2)	6912(1)	49(1)
O(37)	5790(3)	8490(3)	5897(2)	42(1)
O(37')	5841(9)	8861(9)	6149(6)	42(1)
O(37")	5515(9)	8000(9)	5761(4)	42(1)
O(38)	1233(2)	3649(2)	7964(1)	49(1)
N(1)	2759(2)	8947(2)	6898(1)	43(1)
N(2)	2150(2)	5605(2)	5613(1)	49(1)
N(3)	4380(2)	7292(2)	9587(1)	30(1)
N(4)	1520(2)	4664(2)	9929(1)	29(1)
C(1)	2049(3)	8998(3)	7072(2)	55(1)
C(2)	1715(4)	9634(3)	7016(2)	80(2)
C(3)	2125(5)	10226(4)	6778(3)	100(2)
C(4)	2872(4)	10182(3)	6602(2)	87(2)
C(5)	3175(3)	9533(3)	6663(2)	54(1)
C(6)	3980(3)	9415(3)	6484(2)	51(1)
C(7)	4861(4)	10105(3)	6804(2)	74(1)
C(8)	3818(4)	9407(4)	5906(2)	80(2)
C(9)	1337(3)	5005(3)	5551(2)	57(1)
C(10)	808(4)	4551(3)	5070(2)	73(1)
C(11)	1108(4)	4742(4)	4645(2)	86(2)
C(12)	1931(4)	5341(4)	4709(2)	78(2)
C(13)	2461(3)	5771(3)	5202(2)	60(1)
C(14)	3401(4)	6420(3)	5323(2)	64(1)
C(15)	4097(4)	5999(4)	5248(2)	96(2)
C(16)	3408(5)	7091(4)	5001(2)	104(2)
C(17)	4967(2)	7925(2)	9464(1)	37(1)
C(18)	5707(2)	8493(2)	9833(2)	46(1)
C(19)	5855(3)	8402(3)	10342(2)	52(1)
C(20)	5237(2)	7755(2)	10470(1)	43(1)
C(21)	4494(2)	7214(2)	10084(1)	32(1)
C(22)	3730(2)	6534(2)	10183(1)	30(1)
C(23)	3205(2)	6920(2)	10483(1)	41(1)
C(24)	4111(3)	5967(2)	10478(1)	44(1)
C(25)	1212(2)	4947(2)	10310(1)	34(1)
C(26)	1241(2)	4632(2)	10750(1)	40(1)
C(27)	1594(3)	4007(2)	10802(2)	45(1)
C(28)	1882(3)	3701(2)	10404(1)	42(1)
C(29)	1842(2)	4038(2)	9966(1)	32(1)
C(30)	2124(2)	3741(2)	9502(1)	31(1)
C(31)	3066(3)	3643(2)	9664(2)	43(1)
C(32)	1387(3)	2939(2)	9192(2)	47(1)
C(33)	3396(7)	2353(7)	8146(5)	35(2)
C(34)	3117(5)	1533(5)	8176(3)	52(2)
C(35)	3699(7)	1082(5)	8170(4)	65(3)
C(36)	4566(7)	1440(6)	8120(3)	72(2)
C(37)	4872(6)	2274(6)	8098(3)	73(2)
C(38)	4301(5)	2726(4)	8125(3)	53(1)
C(39)	2790(4)	2841(3)	8178(2)	39(1)
C(33')	3583(16)	2516(15)	8064(10)	35(2)
C(34')	3110(11)	1903(9)	8277(7)	52(2)
C(35')	3305(14)	1189(11)	8259(7)	65(3)
C(36')	3921(16)	1083(15)	7993(7)	72(2)
C(37')	4469(11)	1706(11)	7828(7)	73(2)
C(38')	4195(9)	2415(8)	7814(5)	53(1)
C(39')	3278(7)	3251(6)	8056(4)	39(1)
C(41)	5013(3)	9494(2)	8511(2)	41(1)
C(42)	4882(3)	9698(3)	8983(2)	59(1)
C(43)	5556(4)	10382(3)	9341(2)	79(2)
C(44)	6333(4)	10817(3)	9204(2)	81(2)
C(45)	6466(4)	10589(3)	8736(2)	80(2)
C(46)	5809(3)	9948(3)	8388(2)	61(1)
C(47)	4338(2)	8774(2)	8115(1)	34(1)
C(48)	6111(2)	5247(2)	7802(1)	39(1)
C(49)	6095(3)	4658(3)	8096(2)	67(1)
C(50)	6772(4)	4296(4)	8141(3)	92(2)
C(51)	7441(4)	4524(4)	7899(2)	81(2)
C(52)	7486(3)	5137(3)	7627(2)	70(1)
C(53)	6803(3)	5496(3)	7571(2)	57(1)
C(54)	5389(2)	5639(2)	7751(1)	33(1)
C(55)	6766(2)	8159(2)	8778(1)	34(1)
C(56)	7115(2)	7977(2)	9246(2)	45(1)
C(57)	7920(3)	8511(3)	9591(2)	53(1)
C(58)	8381(3)	9236(2)	9472(2)	55(1)
C(59)	8052(3)	9418(3)	9004(2)	68(1)
C(60)	7251(3)	8880(2)	8654(2)	53(1)
C(61)	5871(2)	7579(2)	8413(1)	35(1)
C(62)	4163(7)	4384(5)	6621(4)	38(2)
C(63)	4063(7)	3712(6)	6864(3)	54(2)
C(64)	4644(8)	3266(7)	6852(4)	78(3)
C(65)	5296(9)	3468(8)	6598(5)	84(3)
C(66)	5429(9)	4131(7)	6363(5)	72(2)
C(67)	4882(12)	4601(8)	6391(6)	54(2)
C(62')	4269(14)	4549(11)	6549(7)	38(2)
C(63')	4081(14)	3814(12)	6670(7)	54(2)
C(64')	4568(17)	3271(14)	6568(7)	78(3)
C(65')	5282(19)	3571(19)	6333(10)	84(3)
C(66')	5462(18)	4366(15)	6214(11)	72(2)
C(67')	4880(20)	4811(18)	6302(13)	54(2)
C(68)	3621(2)	4962(2)	6651(1)	40(1)
C(69)	851(9)	7497(13)	5652(7)	53(3)
C(70)	70(20)	7660(40)	5719(17)	99(9)
C(71)	-362(9)	8083(8)	5400(5)	119(2)
C(72)	-56(10)	8132(9)	4941(6)	137(2)
C(73)	748(9)	8013(8)	4870(5)	121(2)
C(74)	1204(7)	7700(8)	5255(5)	87(4)
C(69')	1040(20)	7660(30)	5666(14)	53(3)
C(70')	110(40)	7570(70)	5610(30)	99(9)
C(71')	-367(16)	7848(14)	5215(9)	119(2)
C(72')	239(16)	8483(16)	5037(10)	137(2)
C(73')	1092(15)	8420(14)	4995(9)	121(2)
C(74')	1486(15)	8010(16)	5336(11)	87(4)
C(75)	1383(3)	7177(3)	6053(1)	45(1)
C(76)	2140(2)	8500(2)	8882(1)	31(1)
C(77)	1707(3)	8772(2)	8473(2)	48(1)
C(78)	1525(3)	9497(3)	8556(2)	71(1)
C(79)	1786(3)	9954(3)	9051(2)	69(1)
C(80)	2225(3)	9692(2)	9463(2)	57(1)
C(81)	2407(2)	8964(2)	9380(2)	42(1)
C(82)	2278(2)	7689(2)	8800(1)	27(1)
C(83)	-600(9)	4030(6)	6605(5)	40(2)
C(84)	-726(8)	3214(8)	6416(4)	64(3)
C(85)	-1616(7)	2626(6)	6286(4)	71(2)
C(86)	-2316(9)	2866(8)	6338(5)	70(4)
C(87)	-2212(8)	3663(7)	6531(5)	69(3)
C(88)	-1355(9)	4245(6)	6650(5)	55(2)
C(83')	-558(19)	3775(13)	6625(10)	40(2)
C(84')	-556(16)	3037(16)	6583(8)	64(3)
C(85')	-1387(11)	2361(11)	6384(6)	71(2)
C(86')	-2206(19)	2494(14)	6320(10)	70(4)
C(87')	-2213(19)	3288(17)	6408(13)	69(3)
C(88')	-1385(17)	3910(11)	6561(10)	55(2)
C(89)	327(3)	4623(3)	6772(1)	46(1)
C(90)	-398(2)	7334(2)	7606(1)	38(1)
C(91)	-357(3)	7992(3)	7357(2)	53(1)
C(92)	-803(3)	8528(3)	7477(2)	67(1)
C(93)	-1276(3)	8412(3)	7843(2)	70(1)
C(94)	-1329(3)	7752(3)	8086(2)	69(1)
C(95)	-891(3)	7213(3)	7963(2)	51(1)
C(96)	127(2)	6779(2)	7504(1)	34(1)
C(97)	691(2)	7145(2)	9584(1)	30(1)
C(98)	317(2)	7649(2)	9320(2)	38(1)
C(99)	139(3)	8284(2)	9590(2)	47(1)
C(100)	343(3)	8409(2)	10121(2)	50(1)
C(101)	729(3)	7921(2)	10388(2)	50(1)
C(102)	911(2)	7289(2)	10119(1)	41(1)
C(103)	861(2)	6457(2)	9282(1)	30(1)
C(104)	-932(2)	3502(2)	8222(1)	34(1)
C(105)	-1318(3)	3095(3)	7711(2)	58(1)
C(106)	-2115(4)	2416(3)	7578(2)	86(2)
C(107)	-2556(3)	2160(3)	7941(2)	70(1)
C(108)	-2174(3)	2558(3)	8446(2)	56(1)
C(109)	-1363(3)	3222(2)	8584(2)	44(1)
C(110)	-77(2)	4262(2)	8383(1)	30(1)
C(111)	4707(2)	5408(2)	9136(1)	42(1)
C(112)	6430(3)	7529(4)	7030(3)	92(2)
O(39)	-2526(6)	6340(5)	5548(3)	186(3)
C(113)	-2151(6)	6102(5)	6425(3)	132(3)
C(114)	-2644(10)	6457(9)	6030(4)	244(7)
C(115)	-3123(8)	6539(7)	5145(4)	185(4)
C(116)	-2753(8)	6432(6)	4692(4)	172(4)
________________________________________________________________________________ 
 
Table 3.   Bond lengths [Å] and angles [°] for  ic19479_sq.
_____________________________________________________ 
Mn(1)-O(5) 	1.877(2)
Mn(1)-O(7) 	1.899(2)
Mn(1)-O(15) 	1.968(2)
Mn(1)-O(2) 	1.968(2)
Mn(1)-O(25) 	2.212(2)
Mn(1)-O(1) 	2.271(2)
Mn(1)-Mn(2) 	2.9757(7)
Mn(1)-Mn(4) 	3.0292(6)
Mn(1)-Mn(3) 	3.1404(7)
Mn(2)-O(6) 	1.929(2)
Mn(2)-O(29) 	1.953(2)
Mn(2)-O(23) 	2.004(3)
Mn(2)-O(2) 	2.007(2)
Mn(2)-O(28) 	2.069(3)
Mn(2)-O(5) 	2.083(2)
Mn(2)-Mn(3) 	2.8414(6)
Mn(3)-O(6) 	1.882(2)
Mn(3)-O(8) 	1.923(2)
Mn(3)-O(2) 	1.932(2)
Mn(3)-O(21) 	1.972(2)
Mn(3)-O(27) 	2.186(3)
Mn(3)-O(1) 	2.329(2)
Mn(3)-Mn(4) 	3.1699(7)
Mn(4)-O(7) 	1.880(2)
Mn(4)-O(1) 	1.884(2)
Mn(4)-O(35) 	1.940(2)
Mn(4)-O(17) 	1.970(2)
Mn(4)-O(19) 	2.158(2)
Mn(4)-O(8) 	2.303(2)
Mn(4)-Mn(10) 	3.0942(6)
Mn(5)-O(1) 	1.850(2)
Mn(5)-O(36) 	1.894(2)
Mn(5)-O(3) 	1.930(2)
Mn(5)-O(18) 	2.015(2)
Mn(5)-O(20) 	2.101(3)
Mn(6)-O(5) 	1.849(2)
Mn(6)-O(9) 	1.896(2)
Mn(6)-O(3) 	1.900(2)
Mn(6)-N(1) 	2.020(3)
Mn(6)-O(16) 	2.117(3)
Mn(6)-O(24) 	2.479(3)
Mn(6)-Mn(8) 	3.1085(8)
Mn(6)-Mn(7) 	3.2010(8)
Mn(7)-O(36) 	2.088(3)
Mn(7)-O(9) 	2.142(3)
Mn(7)-O(10) 	2.150(3)
Mn(7)-O(37") 	2.190(8)
Mn(7)-O(37') 	2.253(9)
Mn(7)-O(3) 	2.301(2)
Mn(7)-O(37) 	2.341(4)
Mn(7)-Mn(8) 	3.1947(9)
Mn(8)-O(6) 	1.874(2)
Mn(8)-O(10) 	1.885(3)
Mn(8)-O(3) 	1.913(3)
Mn(8)-N(2) 	2.018(3)
Mn(8)-O(22) 	2.187(3)
Mn(8)-O(24) 	2.301(3)
Mn(9)-O(30) 	2.138(2)
Mn(9)-O(4) 	2.1532(19)
Mn(9)-O(33) 	2.157(2)
Mn(9)-O(31) 	2.195(2)
Mn(9)-O(2) 	2.279(2)
Mn(9)-O(25) 	2.321(2)
Mn(10)-O(7) 	1.873(2)
Mn(10)-O(11) 	1.877(2)
Mn(10)-O(4) 	1.906(2)
Mn(10)-N(3) 	2.039(3)
Mn(10)-O(26) 	2.214(2)
Mn(10)-O(35) 	2.377(2)
Mn(10)-Mn(11) 	2.9803(6)
Mn(11)-O(12) 	1.889(2)
Mn(11)-O(4) 	1.894(2)
Mn(11)-O(32) 	1.942(2)
Mn(11)-N(4) 	2.045(3)
Mn(11)-O(34) 	2.120(2)
Mn(11)-O(11) 	2.277(2)
Mn(11)-Mn(12) 	3.1342(7)
Mn(12)-O(13) 	2.132(2)
Mn(12)-O(12) 	2.134(2)
Mn(12)-O(8) 	2.159(2)
Mn(12)-O(38) 	2.226(3)
Mn(12)-O(4) 	2.269(2)
Mn(12)-O(35) 	2.289(2)
O(9)-C(6) 	1.420(4)
O(10)-C(14) 	1.421(5)
O(11)-C(22) 	1.420(4)
O(12)-C(30) 	1.424(3)
O(13)-C(39') 	1.133(11)
O(13)-C(39) 	1.244(6)
O(15)-C(47) 	1.266(4)
O(14)-C(39) 	1.266(7)
O(14')-C(39') 	1.242(13)
O(16)-C(47) 	1.243(4)
O(17)-C(54) 	1.272(4)
O(18)-C(54) 	1.254(4)
O(19)-C(61) 	1.256(4)
O(20)-C(61) 	1.259(4)
O(21)-C(68) 	1.276(4)
O(22)-C(68) 	1.245(4)
O(23)-C(75) 	1.261(5)
O(24)-C(75) 	1.249(5)
O(25)-C(82) 	1.276(4)
O(26)-C(82) 	1.250(4)
O(27)-C(89) 	1.263(4)
O(28)-C(89) 	1.260(5)
O(29)-C(96) 	1.275(4)
O(30)-C(96) 	1.245(4)
O(31)-C(103) 	1.239(4)
O(32)-C(103) 	1.288(4)
O(33)-C(110) 	1.258(4)
O(34)-C(110) 	1.256(4)
O(35)-C(111) 	1.421(4)
O(36)-C(112) 	1.400(5)
N(1)-C(1) 	1.338(5)
N(1)-C(5) 	1.351(5)
N(2)-C(13) 	1.347(5)
N(2)-C(9) 	1.352(6)
N(3)-C(17) 	1.341(4)
N(3)-C(21) 	1.350(4)
N(4)-C(29) 	1.346(4)
N(4)-C(25) 	1.346(4)
C(1)-C(2) 	1.377(6)
C(2)-C(3) 	1.359(7)
C(3)-C(4) 	1.395(8)
C(4)-C(5) 	1.373(6)
C(5)-C(6) 	1.528(6)
C(6)-C(8) 	1.527(6)
C(6)-C(7) 	1.529(7)
C(9)-C(10) 	1.375(6)
C(10)-C(11) 	1.382(8)
C(11)-C(12) 	1.361(8)
C(12)-C(13) 	1.390(7)
C(13)-C(14) 	1.510(7)
C(14)-C(15) 	1.538(7)
C(14)-C(16) 	1.540(7)
C(17)-C(18) 	1.375(5)
C(18)-C(19) 	1.385(5)
C(19)-C(20) 	1.389(5)
C(20)-C(21) 	1.381(5)
C(21)-C(22) 	1.516(4)
C(22)-C(24) 	1.524(4)
C(22)-C(23) 	1.527(5)
C(25)-C(26) 	1.368(5)
C(26)-C(27) 	1.379(5)
C(27)-C(28) 	1.377(5)
C(28)-C(29) 	1.383(4)
C(29)-C(30) 	1.519(5)
C(30)-C(32) 	1.522(5)
C(30)-C(31) 	1.530(4)
C(33)-C(34) 	1.382(12)
C(33)-C(38) 	1.403(11)
C(33)-C(39) 	1.477(14)
C(34)-C(35) 	1.387(11)
C(35)-C(36) 	1.373(14)
C(36)-C(37) 	1.400(13)
C(37)-C(38) 	1.380(9)
C(33')-C(38') 	1.35(3)
C(33')-C(34') 	1.376(19)
C(33')-C(39') 	1.50(3)
C(34')-C(35') 	1.372(18)
C(35')-C(36') 	1.38(2)
C(36')-C(37') 	1.36(3)
C(37')-C(38') 	1.433(19)
C(41)-C(42) 	1.369(6)
C(41)-C(46) 	1.408(6)
C(41)-C(47) 	1.491(5)
C(42)-C(43) 	1.411(7)
C(43)-C(44) 	1.394(8)
C(44)-C(45) 	1.368(8)
C(45)-C(46) 	1.351(7)
C(48)-C(53) 	1.370(5)
C(48)-C(49) 	1.372(6)
C(48)-C(54) 	1.494(4)
C(49)-C(50) 	1.395(6)
C(50)-C(51) 	1.356(8)
C(51)-C(52) 	1.363(7)
C(52)-C(53) 	1.398(5)
C(55)-C(56) 	1.381(5)
C(55)-C(60) 	1.385(5)
C(55)-C(61) 	1.509(5)
C(56)-C(57) 	1.381(5)
C(57)-C(58) 	1.372(6)
C(58)-C(59) 	1.373(7)
C(59)-C(60) 	1.383(6)
C(62)-C(63) 	1.396(10)
C(62)-C(67) 	1.399(19)
C(62)-C(68) 	1.519(12)
C(63)-C(64) 	1.380(13)
C(64)-C(65) 	1.352(15)
C(65)-C(66) 	1.371(14)
C(66)-C(67) 	1.372(17)
C(62')-C(67') 	1.30(4)
C(62')-C(63') 	1.32(2)
C(62')-C(68) 	1.48(2)
C(63')-C(64') 	1.43(3)
C(64')-C(65') 	1.42(3)
C(65')-C(66') 	1.42(3)
C(66')-C(67') 	1.42(4)
C(69)-C(74) 	1.356(11)
C(69)-C(70) 	1.396(19)
C(69)-C(75) 	1.493(13)
C(70)-C(71) 	1.396(14)
C(71)-C(72) 	1.437(14)
C(72)-C(73) 	1.404(14)
C(73)-C(74) 	1.396(12)
C(69')-C(74') 	1.355(15)
C(69')-C(70') 	1.40(2)
C(69')-C(75) 	1.54(2)
C(70')-C(71') 	1.393(18)
C(71')-C(72') 	1.441(18)
C(72')-C(73') 	1.420(17)
C(73')-C(74') 	1.389(16)
C(76)-C(77) 	1.380(5)
C(76)-C(81) 	1.393(5)
C(76)-C(82) 	1.497(4)
C(77)-C(78) 	1.385(5)
C(78)-C(79) 	1.382(7)
C(79)-C(80) 	1.382(7)
C(80)-C(81) 	1.391(5)
C(83)-C(88) 	1.389(16)
C(83)-C(84) 	1.393(16)
C(83)-C(89) 	1.450(14)
C(84)-C(85) 	1.407(15)
C(85)-C(86) 	1.331(16)
C(86)-C(87) 	1.373(14)
C(87)-C(88) 	1.367(17)
C(83')-C(84') 	1.28(3)
C(83')-C(88') 	1.38(3)
C(83')-C(89) 	1.63(3)
C(84')-C(85') 	1.41(3)
C(85')-C(86') 	1.37(3)
C(86')-C(87') 	1.38(3)
C(87')-C(88') 	1.36(4)
C(90)-C(95) 	1.375(5)
C(90)-C(91) 	1.391(5)
C(90)-C(96) 	1.499(5)
C(91)-C(92) 	1.387(6)
C(92)-C(93) 	1.374(7)
C(93)-C(94) 	1.384(7)
C(94)-C(95) 	1.383(6)
C(97)-C(98) 	1.386(4)
C(97)-C(102) 	1.387(5)
C(97)-C(103) 	1.503(4)
C(98)-C(99) 	1.391(5)
C(99)-C(100) 	1.379(6)
C(100)-C(101) 	1.376(6)
C(101)-C(102) 	1.391(5)
C(104)-C(109) 	1.373(5)
C(104)-C(105) 	1.386(5)
C(104)-C(110) 	1.514(4)
C(105)-C(106) 	1.383(6)
C(106)-C(107) 	1.374(7)
C(107)-C(108) 	1.367(6)
C(108)-C(109) 	1.382(5)
O(39)-C(114) 	1.366(8)
O(39)-C(115) 	1.428(8)
C(113)-C(114) 	1.488(8)
C(115)-C(116) 	1.493(8)

O(5)-Mn(1)-O(7)	173.27(9)
O(5)-Mn(1)-O(15)	94.36(10)
O(7)-Mn(1)-O(15)	86.92(9)
O(5)-Mn(1)-O(2)	85.95(10)
O(7)-Mn(1)-O(2)	93.91(9)
O(15)-Mn(1)-O(2)	170.26(9)
O(5)-Mn(1)-O(25)	100.87(9)
O(7)-Mn(1)-O(25)	85.73(8)
O(15)-Mn(1)-O(25)	89.85(9)
O(2)-Mn(1)-O(25)	80.55(8)
O(5)-Mn(1)-O(1)	98.62(9)
O(7)-Mn(1)-O(1)	74.69(8)
O(15)-Mn(1)-O(1)	106.19(9)
O(2)-Mn(1)-O(1)	83.35(8)
O(25)-Mn(1)-O(1)	153.66(8)
O(5)-Mn(1)-Mn(2)	43.96(7)
O(7)-Mn(1)-Mn(2)	135.80(7)
O(15)-Mn(1)-Mn(2)	137.00(7)
O(2)-Mn(1)-Mn(2)	42.04(6)
O(25)-Mn(1)-Mn(2)	89.07(6)
O(1)-Mn(1)-Mn(2)	92.80(6)
O(5)-Mn(1)-Mn(4)	136.89(7)
O(7)-Mn(1)-Mn(4)	36.49(6)
O(15)-Mn(1)-Mn(4)	100.67(7)
O(2)-Mn(1)-Mn(4)	85.55(6)
O(25)-Mn(1)-Mn(4)	119.16(6)
O(1)-Mn(1)-Mn(4)	38.39(5)
Mn(2)-Mn(1)-Mn(4)	117.09(2)
O(5)-Mn(1)-Mn(3)	87.94(7)
O(7)-Mn(1)-Mn(3)	88.01(7)
O(15)-Mn(1)-Mn(3)	153.72(7)
O(2)-Mn(1)-Mn(3)	35.98(6)
O(25)-Mn(1)-Mn(3)	115.45(6)
O(1)-Mn(1)-Mn(3)	47.72(6)
Mn(2)-Mn(1)-Mn(3)	55.285(15)
Mn(4)-Mn(1)-Mn(3)	61.803(15)
O(6)-Mn(2)-O(29)	177.02(10)
O(6)-Mn(2)-O(23)	94.55(10)
O(29)-Mn(2)-O(23)	82.91(11)
O(6)-Mn(2)-O(2)	82.67(9)
O(29)-Mn(2)-O(2)	99.89(9)
O(23)-Mn(2)-O(2)	177.20(10)
O(6)-Mn(2)-O(28)	88.80(11)
O(29)-Mn(2)-O(28)	92.66(11)
O(23)-Mn(2)-O(28)	88.25(12)
O(2)-Mn(2)-O(28)	91.37(10)
O(6)-Mn(2)-O(5)	92.08(10)
O(29)-Mn(2)-O(5)	86.88(10)
O(23)-Mn(2)-O(5)	100.74(11)
O(2)-Mn(2)-O(5)	79.72(8)
O(28)-Mn(2)-O(5)	170.86(10)
O(6)-Mn(2)-Mn(3)	41.16(6)
O(29)-Mn(2)-Mn(3)	141.66(7)
O(23)-Mn(2)-Mn(3)	134.42(8)
O(2)-Mn(2)-Mn(3)	42.79(6)
O(28)-Mn(2)-Mn(3)	82.02(7)
O(5)-Mn(2)-Mn(3)	92.67(6)
O(6)-Mn(2)-Mn(1)	88.24(7)
O(29)-Mn(2)-Mn(1)	92.69(8)
O(23)-Mn(2)-Mn(1)	139.46(9)
O(2)-Mn(2)-Mn(1)	41.04(6)
O(28)-Mn(2)-Mn(1)	132.27(8)
O(5)-Mn(2)-Mn(1)	38.72(6)
Mn(3)-Mn(2)-Mn(1)	65.301(16)
O(6)-Mn(3)-O(8)	174.14(11)
O(6)-Mn(3)-O(2)	85.97(9)
O(8)-Mn(3)-O(2)	93.23(9)
O(6)-Mn(3)-O(21)	95.49(10)
O(8)-Mn(3)-O(21)	85.04(10)
O(2)-Mn(3)-O(21)	176.89(10)
O(6)-Mn(3)-O(27)	91.46(11)
O(8)-Mn(3)-O(27)	94.33(10)
O(2)-Mn(3)-O(27)	88.73(9)
O(21)-Mn(3)-O(27)	93.97(10)
O(6)-Mn(3)-O(1)	92.21(9)
O(8)-Mn(3)-O(1)	81.94(8)
O(2)-Mn(3)-O(1)	82.61(8)
O(21)-Mn(3)-O(1)	94.58(9)
O(27)-Mn(3)-O(1)	170.33(9)
O(6)-Mn(3)-Mn(2)	42.43(7)
O(8)-Mn(3)-Mn(2)	137.76(7)
O(2)-Mn(3)-Mn(2)	44.90(6)
O(21)-Mn(3)-Mn(2)	137.06(8)
O(27)-Mn(3)-Mn(2)	81.63(7)
O(1)-Mn(3)-Mn(2)	95.10(5)
O(6)-Mn(3)-Mn(1)	84.27(7)
O(8)-Mn(3)-Mn(1)	91.61(7)
O(2)-Mn(3)-Mn(1)	36.77(6)
O(21)-Mn(3)-Mn(1)	140.57(8)
O(27)-Mn(3)-Mn(1)	125.45(7)
O(1)-Mn(3)-Mn(1)	46.19(5)
Mn(2)-Mn(3)-Mn(1)	59.414(16)
O(6)-Mn(3)-Mn(4)	127.99(8)
O(8)-Mn(3)-Mn(4)	46.18(7)
O(2)-Mn(3)-Mn(4)	82.23(6)
O(21)-Mn(3)-Mn(4)	94.72(8)
O(27)-Mn(3)-Mn(4)	138.34(8)
O(1)-Mn(3)-Mn(4)	36.15(5)
Mn(2)-Mn(3)-Mn(4)	116.79(2)
Mn(1)-Mn(3)-Mn(4)	57.373(14)
O(7)-Mn(4)-O(1)	85.19(9)
O(7)-Mn(4)-O(35)	84.52(9)
O(1)-Mn(4)-O(35)	160.89(9)
O(7)-Mn(4)-O(17)	178.11(10)
O(1)-Mn(4)-O(17)	96.47(10)
O(35)-Mn(4)-O(17)	94.14(9)
O(7)-Mn(4)-O(19)	95.37(9)
O(1)-Mn(4)-O(19)	98.37(9)
O(35)-Mn(4)-O(19)	98.60(9)
O(17)-Mn(4)-O(19)	83.50(10)
O(7)-Mn(4)-O(8)	96.58(8)
O(1)-Mn(4)-O(8)	83.45(9)
O(35)-Mn(4)-O(8)	81.79(8)
O(17)-Mn(4)-O(8)	84.53(9)
O(19)-Mn(4)-O(8)	168.02(9)
O(7)-Mn(4)-Mn(1)	36.94(6)
O(1)-Mn(4)-Mn(1)	48.48(6)
O(35)-Mn(4)-Mn(1)	118.61(6)
O(17)-Mn(4)-Mn(1)	144.80(7)
O(19)-Mn(4)-Mn(1)	102.51(6)
O(8)-Mn(4)-Mn(1)	87.61(5)
O(7)-Mn(4)-Mn(10)	34.37(6)
O(1)-Mn(4)-Mn(10)	118.50(7)
O(35)-Mn(4)-Mn(10)	50.18(6)
O(17)-Mn(4)-Mn(10)	144.21(7)
O(19)-Mn(4)-Mn(10)	97.70(7)
O(8)-Mn(4)-Mn(10)	91.74(5)
Mn(1)-Mn(4)-Mn(10)	70.133(15)
O(7)-Mn(4)-Mn(3)	87.47(6)
O(1)-Mn(4)-Mn(3)	46.81(7)
O(35)-Mn(4)-Mn(3)	116.58(7)
O(17)-Mn(4)-Mn(3)	94.33(7)
O(19)-Mn(4)-Mn(3)	144.81(6)
O(8)-Mn(4)-Mn(3)	37.03(5)
Mn(1)-Mn(4)-Mn(3)	60.824(15)
Mn(10)-Mn(4)-Mn(3)	103.898(17)
O(1)-Mn(5)-O(36)	171.18(10)
O(1)-Mn(5)-O(3)	89.60(9)
O(36)-Mn(5)-O(3)	81.84(10)
O(1)-Mn(5)-O(18)	94.83(9)
O(36)-Mn(5)-O(18)	91.68(10)
O(3)-Mn(5)-O(18)	150.98(11)
O(1)-Mn(5)-O(20)	95.86(10)
O(36)-Mn(5)-O(20)	90.46(12)
O(3)-Mn(5)-O(20)	121.44(10)
O(18)-Mn(5)-O(20)	86.67(10)
O(5)-Mn(6)-O(9)	174.08(11)
O(5)-Mn(6)-O(3)	96.83(10)
O(9)-Mn(6)-O(3)	85.56(10)
O(5)-Mn(6)-N(1)	94.95(11)
O(9)-Mn(6)-N(1)	81.58(12)
O(3)-Mn(6)-N(1)	162.83(11)
O(5)-Mn(6)-O(16)	94.60(10)
O(9)-Mn(6)-O(16)	90.38(11)
O(3)-Mn(6)-O(16)	98.40(11)
N(1)-Mn(6)-O(16)	93.05(13)
O(5)-Mn(6)-O(24)	90.42(10)
O(9)-Mn(6)-O(24)	84.61(11)
O(3)-Mn(6)-O(24)	80.78(9)
N(1)-Mn(6)-O(24)	86.70(11)
O(16)-Mn(6)-O(24)	174.97(10)
O(5)-Mn(6)-Mn(8)	86.15(7)
O(9)-Mn(6)-Mn(8)	92.77(9)
O(3)-Mn(6)-Mn(8)	35.51(7)
N(1)-Mn(6)-Mn(8)	133.69(10)
O(16)-Mn(6)-Mn(8)	133.10(8)
O(24)-Mn(6)-Mn(8)	47.00(6)
O(5)-Mn(6)-Mn(7)	141.87(7)
O(9)-Mn(6)-Mn(7)	40.32(8)
O(3)-Mn(6)-Mn(7)	45.25(7)
N(1)-Mn(6)-Mn(7)	121.19(9)
O(16)-Mn(6)-Mn(7)	95.09(8)
O(24)-Mn(6)-Mn(7)	80.79(6)
Mn(8)-Mn(6)-Mn(7)	60.818(19)
O(36)-Mn(7)-O(9)	118.61(11)
O(36)-Mn(7)-O(10)	106.27(11)
O(9)-Mn(7)-O(10)	102.73(11)
O(36)-Mn(7)-O(37")	105.0(4)
O(9)-Mn(7)-O(37")	126.7(4)
O(10)-Mn(7)-O(37")	92.2(4)
O(36)-Mn(7)-O(37')	104.4(4)
O(9)-Mn(7)-O(37')	95.6(4)
O(10)-Mn(7)-O(37')	130.3(4)
O(36)-Mn(7)-O(3)	69.43(9)
O(9)-Mn(7)-O(3)	70.84(9)
O(10)-Mn(7)-O(3)	71.16(9)
O(37")-Mn(7)-O(3)	159.1(4)
O(37')-Mn(7)-O(3)	157.8(4)
O(36)-Mn(7)-O(37)	105.86(13)
O(9)-Mn(7)-O(37)	112.06(14)
O(10)-Mn(7)-O(37)	111.14(15)
O(3)-Mn(7)-O(37)	175.28(12)
O(36)-Mn(7)-Mn(8)	88.48(8)
O(9)-Mn(7)-Mn(8)	85.95(7)
O(10)-Mn(7)-Mn(8)	34.83(6)
O(37")-Mn(7)-Mn(8)	126.2(4)
O(37')-Mn(7)-Mn(8)	164.2(4)
O(3)-Mn(7)-Mn(8)	36.34(6)
O(37)-Mn(7)-Mn(8)	145.89(14)
O(36)-Mn(7)-Mn(6)	94.49(7)
O(9)-Mn(7)-Mn(6)	34.95(6)
O(10)-Mn(7)-Mn(6)	87.05(7)
O(37")-Mn(7)-Mn(6)	159.9(3)
O(37')-Mn(7)-Mn(6)	128.3(4)
O(3)-Mn(7)-Mn(6)	35.90(6)
O(37)-Mn(7)-Mn(6)	146.81(13)
Mn(8)-Mn(7)-Mn(6)	58.160(17)
O(6)-Mn(8)-O(10)	174.15(11)
O(6)-Mn(8)-O(3)	98.43(10)
O(10)-Mn(8)-O(3)	86.11(11)
O(6)-Mn(8)-N(2)	93.87(13)
O(10)-Mn(8)-N(2)	81.37(14)
O(3)-Mn(8)-N(2)	167.12(12)
O(6)-Mn(8)-O(22)	93.22(10)
O(10)-Mn(8)-O(22)	90.70(11)
O(3)-Mn(8)-O(22)	86.90(10)
N(2)-Mn(8)-O(22)	96.25(12)
O(6)-Mn(8)-O(24)	90.22(9)
O(10)-Mn(8)-O(24)	86.47(11)
O(3)-Mn(8)-O(24)	85.37(10)
N(2)-Mn(8)-O(24)	90.80(11)
O(22)-Mn(8)-O(24)	171.93(11)
O(6)-Mn(8)-Mn(6)	87.09(7)
O(10)-Mn(8)-Mn(6)	94.63(9)
O(3)-Mn(8)-Mn(6)	35.24(7)
N(2)-Mn(8)-Mn(6)	142.76(9)
O(22)-Mn(8)-Mn(6)	120.89(8)
O(24)-Mn(8)-Mn(6)	51.97(7)
O(6)-Mn(8)-Mn(7)	143.63(8)
O(10)-Mn(8)-Mn(7)	40.67(9)
O(3)-Mn(8)-Mn(7)	45.46(7)
N(2)-Mn(8)-Mn(7)	121.91(10)
O(22)-Mn(8)-Mn(7)	89.37(8)
O(24)-Mn(8)-Mn(7)	83.55(7)
Mn(6)-Mn(8)-Mn(7)	61.022(19)
O(30)-Mn(9)-O(4)	171.42(9)
O(30)-Mn(9)-O(33)	87.12(9)
O(4)-Mn(9)-O(33)	90.10(8)
O(30)-Mn(9)-O(31)	83.05(9)
O(4)-Mn(9)-O(31)	89.05(8)
O(33)-Mn(9)-O(31)	93.96(9)
O(30)-Mn(9)-O(2)	91.01(8)
O(4)-Mn(9)-O(2)	97.54(7)
O(33)-Mn(9)-O(2)	113.46(9)
O(31)-Mn(9)-O(2)	151.66(8)
O(30)-Mn(9)-O(25)	86.44(8)
O(4)-Mn(9)-O(25)	95.49(8)
O(33)-Mn(9)-O(25)	171.55(8)
O(31)-Mn(9)-O(25)	79.83(8)
O(2)-Mn(9)-O(25)	72.14(8)
O(7)-Mn(10)-O(11)	168.38(9)
O(7)-Mn(10)-O(4)	94.60(9)
O(11)-Mn(10)-O(4)	87.17(9)
O(7)-Mn(10)-N(3)	97.39(10)
O(11)-Mn(10)-N(3)	80.69(10)
O(4)-Mn(10)-N(3)	167.86(10)
O(7)-Mn(10)-O(26)	90.99(8)
O(11)-Mn(10)-O(26)	100.07(9)
O(4)-Mn(10)-O(26)	99.24(8)
N(3)-Mn(10)-O(26)	82.52(9)
O(7)-Mn(10)-O(35)	73.32(8)
O(11)-Mn(10)-O(35)	95.38(8)
O(4)-Mn(10)-O(35)	86.06(8)
N(3)-Mn(10)-O(35)	95.52(9)
O(26)-Mn(10)-O(35)	163.87(8)
O(7)-Mn(10)-Mn(11)	132.78(7)
O(11)-Mn(10)-Mn(11)	49.80(6)
O(4)-Mn(10)-Mn(11)	38.19(6)
N(3)-Mn(10)-Mn(11)	129.77(7)
O(26)-Mn(10)-Mn(11)	96.62(6)
O(35)-Mn(10)-Mn(11)	96.88(5)
O(7)-Mn(10)-Mn(4)	34.52(6)
O(11)-Mn(10)-Mn(4)	134.05(7)
O(4)-Mn(10)-Mn(4)	91.80(6)
N(3)-Mn(10)-Mn(4)	96.98(8)
O(26)-Mn(10)-Mn(4)	125.31(6)
O(35)-Mn(10)-Mn(4)	38.83(5)
Mn(11)-Mn(10)-Mn(4)	121.98(2)
O(12)-Mn(11)-O(4)	87.07(9)
O(12)-Mn(11)-O(32)	169.09(9)
O(4)-Mn(11)-O(32)	100.74(9)
O(12)-Mn(11)-N(4)	80.96(10)
O(4)-Mn(11)-N(4)	165.75(9)
O(32)-Mn(11)-N(4)	90.19(10)
O(12)-Mn(11)-O(34)	96.36(10)
O(4)-Mn(11)-O(34)	96.54(9)
O(32)-Mn(11)-O(34)	90.35(9)
N(4)-Mn(11)-O(34)	92.46(10)
O(12)-Mn(11)-O(11)	88.35(9)
O(4)-Mn(11)-O(11)	76.81(8)
O(32)-Mn(11)-O(11)	86.07(9)
N(4)-Mn(11)-O(11)	95.04(9)
O(34)-Mn(11)-O(11)	171.69(8)
O(12)-Mn(11)-Mn(10)	92.72(7)
O(4)-Mn(11)-Mn(10)	38.49(6)
O(32)-Mn(11)-Mn(10)	88.84(7)
N(4)-Mn(11)-Mn(10)	134.00(8)
O(34)-Mn(11)-Mn(10)	133.53(6)
O(11)-Mn(11)-Mn(10)	39.02(5)
O(12)-Mn(11)-Mn(12)	41.73(6)
O(4)-Mn(11)-Mn(12)	45.91(6)
O(32)-Mn(11)-Mn(12)	146.65(7)
N(4)-Mn(11)-Mn(12)	122.69(7)
O(34)-Mn(11)-Mn(12)	93.38(6)
O(11)-Mn(11)-Mn(12)	85.58(6)
Mn(10)-Mn(11)-Mn(12)	64.975(16)
O(13)-Mn(12)-O(12)	100.05(10)
O(13)-Mn(12)-O(8)	93.02(10)
O(12)-Mn(12)-O(8)	166.91(8)
O(13)-Mn(12)-O(38)	87.59(10)
O(12)-Mn(12)-O(38)	96.00(10)
O(8)-Mn(12)-O(38)	85.29(10)
O(13)-Mn(12)-O(4)	169.66(9)
O(12)-Mn(12)-O(4)	72.49(7)
O(8)-Mn(12)-O(4)	94.44(8)
O(38)-Mn(12)-O(4)	100.15(8)
O(13)-Mn(12)-O(35)	94.20(9)
O(12)-Mn(12)-O(35)	100.35(8)
O(8)-Mn(12)-O(35)	77.70(8)
O(38)-Mn(12)-O(35)	162.97(10)
O(4)-Mn(12)-O(35)	80.43(7)
O(13)-Mn(12)-Mn(11)	136.15(8)
O(12)-Mn(12)-Mn(11)	36.11(5)
O(8)-Mn(12)-Mn(11)	130.82(6)
O(38)-Mn(12)-Mn(11)	95.73(7)
O(4)-Mn(12)-Mn(11)	36.82(5)
O(35)-Mn(12)-Mn(11)	94.67(6)
Mn(5)-O(1)-Mn(4)	120.77(11)
Mn(5)-O(1)-Mn(1)	122.87(11)
Mn(4)-O(1)-Mn(1)	93.13(9)
Mn(5)-O(1)-Mn(3)	127.44(11)
Mn(4)-O(1)-Mn(3)	97.04(9)
Mn(1)-O(1)-Mn(3)	86.10(7)
Mn(3)-O(2)-Mn(1)	107.26(10)
Mn(3)-O(2)-Mn(2)	92.31(9)
Mn(1)-O(2)-Mn(2)	96.92(9)
Mn(3)-O(2)-Mn(9)	132.83(10)
Mn(1)-O(2)-Mn(9)	104.33(9)
Mn(2)-O(2)-Mn(9)	117.65(9)
Mn(6)-O(3)-Mn(8)	109.25(11)
Mn(6)-O(3)-Mn(5)	127.34(13)
Mn(8)-O(3)-Mn(5)	116.11(12)
Mn(6)-O(3)-Mn(7)	98.85(10)
Mn(8)-O(3)-Mn(7)	98.20(9)
Mn(5)-O(3)-Mn(7)	99.73(9)
Mn(11)-O(4)-Mn(10)	103.32(10)
Mn(11)-O(4)-Mn(9)	116.23(9)
Mn(10)-O(4)-Mn(9)	115.81(9)
Mn(11)-O(4)-Mn(12)	97.27(8)
Mn(10)-O(4)-Mn(12)	103.50(8)
Mn(9)-O(4)-Mn(12)	118.02(9)
Mn(6)-O(5)-Mn(1)	121.64(12)
Mn(6)-O(5)-Mn(2)	124.05(12)
Mn(1)-O(5)-Mn(2)	97.31(10)
Mn(8)-O(6)-Mn(3)	125.09(12)
Mn(8)-O(6)-Mn(2)	125.96(13)
Mn(3)-O(6)-Mn(2)	96.41(10)
Mn(10)-O(7)-Mn(4)	111.10(10)
Mn(10)-O(7)-Mn(1)	137.75(11)
Mn(4)-O(7)-Mn(1)	106.57(10)
Mn(3)-O(8)-Mn(12)	147.96(11)
Mn(3)-O(8)-Mn(4)	96.79(9)
Mn(12)-O(8)-Mn(4)	95.98(8)
C(6)-O(9)-Mn(6)	119.5(2)
C(6)-O(9)-Mn(7)	135.7(2)
Mn(6)-O(9)-Mn(7)	104.73(11)
C(14)-O(10)-Mn(8)	119.8(3)
C(14)-O(10)-Mn(7)	135.7(3)
Mn(8)-O(10)-Mn(7)	104.50(12)
C(22)-O(11)-Mn(10)	120.85(18)
C(22)-O(11)-Mn(11)	143.59(18)
Mn(10)-O(11)-Mn(11)	91.17(8)
C(30)-O(12)-Mn(11)	120.02(18)
C(30)-O(12)-Mn(12)	136.91(18)
Mn(11)-O(12)-Mn(12)	102.17(9)
C(39')-O(13)-Mn(12)	134.5(5)
C(39)-O(13)-Mn(12)	133.1(3)
C(47)-O(15)-Mn(1)	129.6(2)
C(47)-O(16)-Mn(6)	129.8(2)
C(54)-O(17)-Mn(4)	124.2(2)
C(54)-O(18)-Mn(5)	135.0(2)
C(61)-O(19)-Mn(4)	129.3(2)
C(61)-O(20)-Mn(5)	126.2(2)
C(68)-O(21)-Mn(3)	125.8(2)
C(68)-O(22)-Mn(8)	130.2(2)
C(75)-O(23)-Mn(2)	123.2(2)
C(75)-O(24)-Mn(8)	125.9(3)
C(75)-O(24)-Mn(6)	120.2(2)
Mn(8)-O(24)-Mn(6)	81.04(8)
C(82)-O(25)-Mn(1)	118.85(18)
C(82)-O(25)-Mn(9)	120.69(18)
Mn(1)-O(25)-Mn(9)	95.64(8)
C(82)-O(26)-Mn(10)	121.6(2)
C(89)-O(27)-Mn(3)	122.4(3)
C(89)-O(28)-Mn(2)	127.7(2)
C(96)-O(29)-Mn(2)	129.3(2)
C(96)-O(30)-Mn(9)	125.8(2)
C(103)-O(31)-Mn(9)	135.1(2)
C(103)-O(32)-Mn(11)	127.3(2)
C(110)-O(33)-Mn(9)	125.6(2)
C(110)-O(34)-Mn(11)	133.2(2)
C(111)-O(35)-Mn(4)	121.08(19)
C(111)-O(35)-Mn(12)	120.6(2)
Mn(4)-O(35)-Mn(12)	102.99(9)
C(111)-O(35)-Mn(10)	124.2(2)
Mn(4)-O(35)-Mn(10)	90.99(8)
Mn(12)-O(35)-Mn(10)	89.55(7)
C(112)-O(36)-Mn(5)	123.3(3)
C(112)-O(36)-Mn(7)	127.5(3)
Mn(5)-O(36)-Mn(7)	108.98(11)
C(1)-N(1)-C(5)	120.0(3)
C(1)-N(1)-Mn(6)	125.8(3)
C(5)-N(1)-Mn(6)	114.2(3)
C(13)-N(2)-C(9)	120.3(4)
C(13)-N(2)-Mn(8)	114.2(3)
C(9)-N(2)-Mn(8)	125.4(3)
C(17)-N(3)-C(21)	120.0(3)
C(17)-N(3)-Mn(10)	125.5(2)
C(21)-N(3)-Mn(10)	114.3(2)
C(29)-N(4)-C(25)	120.0(3)
C(29)-N(4)-Mn(11)	113.8(2)
C(25)-N(4)-Mn(11)	125.8(2)
N(1)-C(1)-C(2)	122.2(4)
C(3)-C(2)-C(1)	118.5(5)
C(2)-C(3)-C(4)	119.7(5)
C(5)-C(4)-C(3)	119.7(4)
N(1)-C(5)-C(4)	120.0(4)
N(1)-C(5)-C(6)	115.6(3)
C(4)-C(5)-C(6)	124.4(4)
O(9)-C(6)-C(8)	108.3(4)
O(9)-C(6)-C(5)	108.2(3)
C(8)-C(6)-C(5)	110.4(4)
O(9)-C(6)-C(7)	110.0(3)
C(8)-C(6)-C(7)	111.8(4)
C(5)-C(6)-C(7)	108.2(4)
N(2)-C(9)-C(10)	121.3(5)
C(9)-C(10)-C(11)	118.5(5)
C(12)-C(11)-C(10)	120.1(5)
C(11)-C(12)-C(13)	119.8(5)
N(2)-C(13)-C(12)	119.9(5)
N(2)-C(13)-C(14)	115.4(4)
C(12)-C(13)-C(14)	124.6(4)
O(10)-C(14)-C(13)	108.2(3)
O(10)-C(14)-C(15)	108.1(4)
C(13)-C(14)-C(15)	108.8(5)
O(10)-C(14)-C(16)	107.1(4)
C(13)-C(14)-C(16)	112.3(4)
C(15)-C(14)-C(16)	112.2(5)
N(3)-C(17)-C(18)	121.6(3)
C(17)-C(18)-C(19)	119.1(3)
C(18)-C(19)-C(20)	119.2(3)
C(21)-C(20)-C(19)	119.1(3)
N(3)-C(21)-C(20)	121.0(3)
N(3)-C(21)-C(22)	115.2(3)
C(20)-C(21)-C(22)	123.7(3)
O(11)-C(22)-C(21)	107.9(2)
O(11)-C(22)-C(24)	108.6(3)
C(21)-C(22)-C(24)	111.2(3)
O(11)-C(22)-C(23)	109.5(3)
C(21)-C(22)-C(23)	108.9(3)
C(24)-C(22)-C(23)	110.8(3)
N(4)-C(25)-C(26)	121.8(3)
C(25)-C(26)-C(27)	118.9(3)
C(28)-C(27)-C(26)	119.2(3)
C(27)-C(28)-C(29)	120.0(3)
N(4)-C(29)-C(28)	120.0(3)
N(4)-C(29)-C(30)	115.4(3)
C(28)-C(29)-C(30)	124.5(3)
O(12)-C(30)-C(29)	107.8(2)
O(12)-C(30)-C(32)	109.2(3)
C(29)-C(30)-C(32)	109.2(3)
O(12)-C(30)-C(31)	107.8(3)
C(29)-C(30)-C(31)	111.5(3)
C(32)-C(30)-C(31)	111.3(3)
C(34)-C(33)-C(38)	117.8(10)
C(34)-C(33)-C(39)	121.2(7)
C(38)-C(33)-C(39)	120.8(8)
C(33)-C(34)-C(35)	121.4(9)
C(36)-C(35)-C(34)	120.4(8)
C(35)-C(36)-C(37)	119.3(7)
C(38)-C(37)-C(36)	119.9(8)
C(37)-C(38)-C(33)	121.0(8)
O(13)-C(39)-O(14)	123.3(5)
O(13)-C(39)-C(33)	119.8(6)
O(14)-C(39)-C(33)	116.6(6)
C(38')-C(33')-C(34')	122(2)
C(38')-C(33')-C(39')	121.4(14)
C(34')-C(33')-C(39')	116.4(18)
C(35')-C(34')-C(33')	119(2)
C(34')-C(35')-C(36')	119(2)
C(37')-C(36')-C(35')	123(2)
C(36')-C(37')-C(38')	115.3(14)
C(33')-C(38')-C(37')	120.1(15)
O(13)-C(39')-O(14')	118.0(9)
O(13)-C(39')-C(33')	124.7(12)
O(14')-C(39')-C(33')	117.0(12)
C(42)-C(41)-C(46)	120.8(4)
C(42)-C(41)-C(47)	121.6(4)
C(46)-C(41)-C(47)	117.6(4)
C(41)-C(42)-C(43)	118.5(5)
C(44)-C(43)-C(42)	118.9(5)
C(45)-C(44)-C(43)	121.8(5)
C(46)-C(45)-C(44)	119.3(5)
C(45)-C(46)-C(41)	120.7(5)
O(16)-C(47)-O(15)	125.6(3)
O(16)-C(47)-C(41)	117.7(3)
O(15)-C(47)-C(41)	116.6(3)
C(53)-C(48)-C(49)	120.2(3)
C(53)-C(48)-C(54)	120.1(3)
C(49)-C(48)-C(54)	119.6(3)
C(48)-C(49)-C(50)	119.2(4)
C(51)-C(50)-C(49)	120.5(5)
C(50)-C(51)-C(52)	120.5(4)
C(51)-C(52)-C(53)	119.6(4)
C(48)-C(53)-C(52)	119.9(4)
O(18)-C(54)-O(17)	125.5(3)
O(18)-C(54)-C(48)	117.6(3)
O(17)-C(54)-C(48)	116.9(3)
C(56)-C(55)-C(60)	118.7(3)
C(56)-C(55)-C(61)	120.1(3)
C(60)-C(55)-C(61)	121.2(3)
C(55)-C(56)-C(57)	120.9(4)
C(58)-C(57)-C(56)	120.0(4)
C(57)-C(58)-C(59)	119.8(4)
C(58)-C(59)-C(60)	120.4(4)
C(59)-C(60)-C(55)	120.2(4)
O(19)-C(61)-O(20)	126.2(3)
O(19)-C(61)-C(55)	116.6(3)
O(20)-C(61)-C(55)	117.2(3)
C(63)-C(62)-C(67)	118.0(9)
C(63)-C(62)-C(68)	124.2(8)
C(67)-C(62)-C(68)	117.3(6)
C(64)-C(63)-C(62)	119.8(9)
C(65)-C(64)-C(63)	120.2(9)
C(64)-C(65)-C(66)	122.0(11)
C(65)-C(66)-C(67)	118.3(10)
C(66)-C(67)-C(62)	121.4(8)
C(67')-C(62')-C(63')	122(2)
C(67')-C(62')-C(68)	124.9(16)
C(63')-C(62')-C(68)	112.7(16)
C(62')-C(63')-C(64')	122.9(18)
C(65')-C(64')-C(63')	116.0(19)
C(64')-C(65')-C(66')	119(2)
C(67')-C(66')-C(65')	119(2)
C(62')-C(67')-C(66')	121.0(18)
O(22)-C(68)-O(21)	123.8(3)
O(22)-C(68)-C(62')	109.2(7)
O(21)-C(68)-C(62')	127.0(7)
O(22)-C(68)-C(62)	123.8(4)
O(21)-C(68)-C(62)	112.4(4)
C(74)-C(69)-C(70)	121.8(10)
C(74)-C(69)-C(75)	117.6(12)
C(70)-C(69)-C(75)	120.2(13)
C(71)-C(70)-C(69)	122.4(13)
C(70)-C(71)-C(72)	111.4(17)
C(73)-C(72)-C(71)	126.1(13)
C(74)-C(73)-C(72)	115.4(12)
C(69)-C(74)-C(73)	120.6(11)
C(74')-C(69')-C(70')	121.3(17)
C(74')-C(69')-C(75)	125(2)
C(70')-C(69')-C(75)	112.2(19)
C(71')-C(70')-C(69')	120.4(19)
C(70')-C(71')-C(72')	112(3)
C(73')-C(72')-C(71')	119.1(18)
C(74')-C(73')-C(72')	116.2(17)
C(69')-C(74')-C(73')	119.9(17)
O(24)-C(75)-O(23)	125.0(3)
O(24)-C(75)-C(69)	122.3(6)
O(23)-C(75)-C(69)	112.6(6)
O(24)-C(75)-C(69')	111.5(11)
O(23)-C(75)-C(69')	123.3(11)
C(77)-C(76)-C(81)	119.7(3)
C(77)-C(76)-C(82)	120.8(3)
C(81)-C(76)-C(82)	119.4(3)
C(76)-C(77)-C(78)	120.6(4)
C(79)-C(78)-C(77)	119.7(4)
C(78)-C(79)-C(80)	120.4(4)
C(79)-C(80)-C(81)	119.9(4)
C(80)-C(81)-C(76)	119.8(4)
O(26)-C(82)-O(25)	124.4(3)
O(26)-C(82)-C(76)	118.7(3)
O(25)-C(82)-C(76)	116.9(3)
C(88)-C(83)-C(84)	119.4(11)
C(88)-C(83)-C(89)	121.9(8)
C(84)-C(83)-C(89)	118.7(10)
C(83)-C(84)-C(85)	118.9(11)
C(86)-C(85)-C(84)	119.3(10)
C(85)-C(86)-C(87)	123.2(12)
C(88)-C(87)-C(86)	118.3(10)
C(87)-C(88)-C(83)	120.8(9)
C(84')-C(83')-C(88')	119(2)
C(84')-C(83')-C(89)	128(2)
C(88')-C(83')-C(89)	113.0(14)
C(83')-C(84')-C(85')	121(2)
C(86')-C(85')-C(84')	119.2(19)
C(85')-C(86')-C(87')	120(3)
C(88')-C(87')-C(86')	118(3)
C(87')-C(88')-C(83')	122.7(19)
O(28)-C(89)-O(27)	125.7(4)
O(28)-C(89)-C(83)	110.2(4)
O(27)-C(89)-C(83)	124.2(5)
O(28)-C(89)-C(83')	126.5(7)
O(27)-C(89)-C(83')	107.8(8)
C(95)-C(90)-C(91)	119.8(3)
C(95)-C(90)-C(96)	119.8(3)
C(91)-C(90)-C(96)	120.3(3)
C(92)-C(91)-C(90)	119.7(4)
C(93)-C(92)-C(91)	120.0(4)
C(92)-C(93)-C(94)	120.5(4)
C(95)-C(94)-C(93)	119.5(5)
C(90)-C(95)-C(94)	120.5(4)
O(30)-C(96)-O(29)	125.2(3)
O(30)-C(96)-C(90)	119.3(3)
O(29)-C(96)-C(90)	115.5(3)
C(98)-C(97)-C(102)	119.7(3)
C(98)-C(97)-C(103)	118.8(3)
C(102)-C(97)-C(103)	121.5(3)
C(97)-C(98)-C(99)	120.0(4)
C(100)-C(99)-C(98)	119.7(3)
C(101)-C(100)-C(99)	120.7(3)
C(100)-C(101)-C(102)	119.7(4)
C(97)-C(102)-C(101)	120.2(3)
O(31)-C(103)-O(32)	125.6(3)
O(31)-C(103)-C(97)	118.7(3)
O(32)-C(103)-C(97)	115.7(3)
C(109)-C(104)-C(105)	118.6(3)
C(109)-C(104)-C(110)	119.7(3)
C(105)-C(104)-C(110)	121.6(3)
C(106)-C(105)-C(104)	119.7(4)
C(107)-C(106)-C(105)	121.0(4)
C(108)-C(107)-C(106)	119.3(4)
C(107)-C(108)-C(109)	119.9(4)
C(104)-C(109)-C(108)	121.3(4)
O(34)-C(110)-O(33)	126.4(3)
O(34)-C(110)-C(104)	115.0(3)
O(33)-C(110)-C(104)	118.5(3)
C(114)-O(39)-C(115)	117.4(9)
O(39)-C(114)-C(113)	117.1(10)
O(39)-C(115)-C(116)	103.9(9)

_____________________________________________________________
Symmetry transformations used to generate equivalent atoms: 
 

 Table 4.   Anisotropic displacement parameters  (Å2x 103) for ic19479_sq.  The anisotropic
displacement factor exponent takes the form:  -2p2[ h2 a*2U11 + ...  + 2 h k a* b* U12 ]
______________________________________________________________________________ 
	U11	U22 	U33	U23	U13	U12
______________________________________________________________________________ 
Mn(1)	27(1) 	28(1)	25(1) 	9(1)	7(1) 	12(1)
Mn(2)	31(1) 	44(1)	29(1) 	9(1)	7(1) 	21(1)
Mn(3)	32(1) 	33(1)	26(1) 	5(1)	6(1) 	16(1)
Mn(4)	24(1) 	32(1)	29(1) 	11(1)	7(1) 	13(1)
Mn(5)	30(1) 	47(1)	37(1) 	19(1)	14(1) 	18(1)
Mn(6)	40(1) 	42(1)	35(1) 	20(1)	15(1) 	22(1)
Mn(7)	56(1) 	67(1)	49(1) 	32(1)	29(1) 	33(1)
Mn(8)	43(1) 	56(1)	26(1) 	11(1)	12(1) 	25(1)
Mn(9)	24(1) 	28(1)	28(1) 	7(1)	4(1) 	11(1)
Mn(10)	23(1) 	27(1)	25(1) 	7(1)	3(1) 	8(1)
Mn(11)	25(1) 	27(1)	28(1) 	7(1)	7(1) 	12(1)
Mn(12)	32(1) 	28(1)	32(1) 	8(1)	10(1) 	14(1)
O(1)	28(1) 	37(1)	31(1) 	12(1)	9(1) 	16(1)
O(2)	26(1) 	30(1)	28(1) 	4(1)	7(1) 	12(1)
O(3)	37(1) 	48(1)	31(1) 	16(1)	12(1) 	23(1)
O(4)	22(1) 	26(1)	29(1) 	6(1)	5(1) 	9(1)
O(5)	35(1) 	38(1)	32(1) 	14(1)	12(1) 	19(1)
O(6)	35(1) 	47(1)	25(1) 	8(1)	6(1) 	20(1)
O(7)	26(1) 	30(1)	28(1) 	10(1)	7(1) 	13(1)
O(8)	31(1) 	34(1)	30(1) 	10(1)	8(1) 	16(1)
O(9)	50(2) 	51(2)	47(2) 	27(1)	23(1) 	24(1)
O(10)	61(2) 	72(2)	34(1) 	20(1)	25(1) 	33(2)
O(11)	26(1) 	32(1)	25(1) 	7(1)	2(1) 	8(1)
O(12)	33(1) 	29(1)	33(1) 	12(1)	13(1) 	16(1)
O(13)	46(1) 	38(1)	61(2) 	1(1)	8(1) 	24(1)
O(15)	39(1) 	29(1)	33(1) 	9(1)	11(1) 	8(1)
O(14)	59(3) 	48(2)	97(4) 	6(2)	20(2) 	28(2)
O(14')	59(3) 	48(2)	97(4) 	6(2)	20(2) 	28(2)
O(16)	46(2) 	54(2)	40(1) 	19(1)	13(1) 	7(1)
O(17)	33(1) 	41(1)	46(1) 	15(1)	15(1) 	21(1)
O(18)	34(1) 	45(1)	42(1) 	15(1)	16(1) 	22(1)
O(19)	26(1) 	41(1)	45(1) 	15(1)	8(1) 	8(1)
O(20)	41(1) 	39(1)	42(1) 	11(1)	10(1) 	14(1)
O(21)	45(1) 	40(1)	42(1) 	4(1)	15(1) 	21(1)
O(22)	50(2) 	68(2)	46(2) 	22(1)	22(1) 	34(1)
O(23)	45(2) 	76(2)	50(2) 	22(2)	14(1) 	33(2)
O(24)	46(2) 	59(2)	39(1) 	15(1)	8(1) 	26(1)
O(25)	30(1) 	29(1)	30(1) 	6(1)	8(1) 	12(1)
O(26)	34(1) 	36(1)	31(1) 	8(1)	7(1) 	16(1)
O(27)	34(1) 	42(1)	56(2) 	-3(1)	4(1) 	11(1)
O(28)	37(1) 	70(2)	42(2) 	5(1)	3(1) 	25(1)
O(29)	36(1) 	47(1)	45(1) 	16(1)	13(1) 	23(1)
O(30)	30(1) 	43(1)	38(1) 	12(1)	3(1) 	16(1)
O(31)	30(1) 	40(1)	32(1) 	8(1)	7(1) 	17(1)
O(32)	34(1) 	30(1)	33(1) 	6(1)	9(1) 	17(1)
O(33)	34(1) 	33(1)	38(1) 	9(1)	6(1) 	6(1)
O(34)	27(1) 	39(1)	33(1) 	4(1)	4(1) 	8(1)
O(35)	24(1) 	35(1)	32(1) 	12(1)	4(1) 	14(1)
O(36)	36(1) 	71(2)	54(2) 	33(1)	22(1) 	23(1)
O(37)	55(2) 	60(3)	28(2) 	25(2)	31(2) 	23(2)
O(37')	55(2) 	60(3)	28(2) 	25(2)	31(2) 	23(2)
O(37")	55(2) 	60(3)	28(2) 	25(2)	31(2) 	23(2)
O(38)	37(1) 	42(2)	63(2) 	-2(1)	7(1) 	16(1)
N(1)	50(2) 	45(2)	47(2) 	22(2)	18(2) 	26(2)
N(2)	60(2) 	65(2)	29(2) 	6(2)	11(2) 	35(2)
N(3)	24(1) 	31(1)	28(1) 	6(1)	4(1) 	7(1)
N(4)	25(1) 	29(1)	33(1) 	8(1)	8(1) 	9(1)
C(1)	58(2) 	52(2)	73(3) 	28(2)	28(2) 	31(2)
C(2)	86(4) 	76(3)	119(5) 	48(3)	56(4) 	56(3)
C(3)	120(5) 	90(4)	150(6) 	76(4)	68(5) 	77(4)
C(4)	115(5) 	71(3)	125(5) 	67(4)	68(4) 	61(3)
C(5)	65(3) 	54(2)	60(3) 	33(2)	25(2) 	31(2)
C(6)	62(2) 	53(2)	51(2) 	32(2)	22(2) 	26(2)
C(7)	77(3) 	58(3)	94(4) 	27(3)	39(3) 	15(3)
C(8)	104(4) 	104(4)	66(3) 	56(3)	38(3) 	57(4)
C(9)	58(3) 	69(3)	40(2) 	-3(2)	8(2) 	25(2)
C(10)	70(3) 	88(4)	48(3) 	-12(2)	4(2) 	29(3)
C(11)	90(4) 	119(5)	38(3) 	-18(3)	-4(3) 	48(4)
C(12)	93(4) 	112(5)	33(2) 	6(3)	16(2) 	46(4)
C(13)	76(3) 	89(3)	32(2) 	15(2)	18(2) 	49(3)
C(14)	82(3) 	92(4)	31(2) 	18(2)	27(2) 	40(3)
C(15)	91(4) 	134(6)	76(4) 	-9(4)	46(3) 	51(4)
C(16)	135(6) 	125(5)	48(3) 	41(3)	24(3) 	31(5)
C(17)	33(2) 	38(2)	36(2) 	10(2)	9(1) 	6(1)
C(18)	34(2) 	43(2)	47(2) 	3(2)	8(2) 	-3(2)
C(19)	33(2) 	57(2)	44(2) 	2(2)	1(2) 	-4(2)
C(20)	34(2) 	51(2)	32(2) 	6(2)	1(2) 	5(2)
C(21)	29(2) 	36(2)	30(2) 	6(1)	5(1) 	12(1)
C(22)	28(2) 	33(2)	27(2) 	7(1)	4(1) 	8(1)
C(23)	36(2) 	40(2)	41(2) 	1(2)	14(2) 	6(2)
C(24)	48(2) 	47(2)	34(2) 	14(2)	0(2) 	18(2)
C(25)	30(2) 	34(2)	40(2) 	9(1)	14(1) 	10(1)
C(26)	41(2) 	41(2)	41(2) 	9(2)	20(2) 	10(2)
C(27)	51(2) 	50(2)	44(2) 	22(2)	23(2) 	18(2)
C(28)	53(2) 	42(2)	46(2) 	22(2)	22(2) 	24(2)
C(29)	29(2) 	32(2)	37(2) 	11(1)	11(1) 	10(1)
C(30)	35(2) 	28(2)	36(2) 	13(1)	13(1) 	14(1)
C(31)	49(2) 	54(2)	45(2) 	21(2)	19(2) 	33(2)
C(32)	57(2) 	33(2)	48(2) 	7(2)	18(2) 	9(2)
C(33)	32(5) 	27(6)	45(5) 	6(3)	10(3) 	9(4)
C(34)	51(3) 	36(5)	71(5) 	12(4)	6(3) 	25(4)
C(35)	97(9) 	38(4)	64(5) 	5(4)	12(5) 	36(5)
C(36)	92(6) 	82(6)	58(5) 	0(4)	9(5) 	68(6)
C(37)	67(5) 	93(6)	73(5) 	9(4)	23(4) 	49(5)
C(38)	60(3) 	54(4)	52(4) 	11(3)	17(3) 	30(3)
C(39)	41(3) 	33(3)	45(3) 	9(2)	9(2) 	16(2)
C(33')	32(5) 	27(6)	45(5) 	6(3)	10(3) 	9(4)
C(34')	51(3) 	36(5)	71(5) 	12(4)	6(3) 	25(4)
C(35')	97(9) 	38(4)	64(5) 	5(4)	12(5) 	36(5)
C(36')	92(6) 	82(6)	58(5) 	0(4)	9(5) 	68(6)
C(37')	67(5) 	93(6)	73(5) 	9(4)	23(4) 	49(5)
C(38')	60(3) 	54(4)	52(4) 	11(3)	17(3) 	30(3)
C(39')	41(3) 	33(3)	45(3) 	9(2)	9(2) 	16(2)
C(41)	46(2) 	26(2)	48(2) 	13(2)	4(2) 	14(2)
C(42)	63(3) 	50(2)	57(3) 	0(2)	4(2) 	22(2)
C(43)	103(4) 	64(3)	58(3) 	-12(2)	-4(3) 	39(3)
C(44)	85(4) 	26(2)	98(4) 	6(2)	-11(3) 	0(2)
C(45)	81(4) 	50(3)	81(4) 	23(3)	4(3) 	-5(3)
C(46)	58(3) 	43(2)	68(3) 	21(2)	8(2) 	0(2)
C(47)	32(2) 	32(2)	42(2) 	14(1)	10(1) 	15(1)
C(48)	31(2) 	46(2)	45(2) 	9(2)	9(2) 	21(2)
C(49)	63(3) 	75(3)	93(4) 	42(3)	35(3) 	46(3)
C(50)	100(4) 	113(5)	121(5) 	67(4)	49(4) 	87(4)
C(51)	77(3) 	114(5)	86(4) 	25(3)	23(3) 	77(4)
C(52)	52(3) 	103(4)	77(3) 	18(3)	29(2) 	50(3)
C(53)	52(2) 	68(3)	70(3) 	22(2)	28(2) 	36(2)
C(54)	30(2) 	38(2)	35(2) 	6(1)	8(1) 	17(1)
C(55)	28(2) 	33(2)	46(2) 	6(1)	14(1) 	14(1)
C(56)	31(2) 	42(2)	60(2) 	15(2)	6(2) 	12(2)
C(57)	36(2) 	56(2)	59(3) 	11(2)	-1(2) 	18(2)
C(58)	40(2) 	39(2)	73(3) 	-8(2)	4(2) 	12(2)
C(59)	64(3) 	37(2)	86(4) 	6(2)	15(3) 	-1(2)
C(60)	55(2) 	41(2)	55(2) 	12(2)	12(2) 	7(2)
C(61)	30(2) 	37(2)	45(2) 	9(2)	13(2) 	17(1)
C(62)	39(2) 	38(3)	41(3) 	8(2)	13(2) 	17(2)
C(63)	67(3) 	53(4)	62(6) 	31(4)	32(5) 	34(3)
C(64)	95(5) 	77(4)	91(8) 	32(7)	36(7) 	58(4)
C(65)	77(4) 	85(6)	121(12) 	21(8)	52(8) 	53(4)
C(66)	71(4) 	67(8)	113(10) 	38(4)	53(5) 	44(5)
C(67)	60(3) 	55(8)	65(7) 	22(4)	26(4) 	37(5)
C(62')	39(2) 	38(3)	41(3) 	8(2)	13(2) 	17(2)
C(63')	67(3) 	53(4)	62(6) 	31(4)	32(5) 	34(3)
C(64')	95(5) 	77(4)	91(8) 	32(7)	36(7) 	58(4)
C(65')	77(4) 	85(6)	121(12) 	21(8)	52(8) 	53(4)
C(66')	71(4) 	67(8)	113(10) 	38(4)	53(5) 	44(5)
C(67')	60(3) 	55(8)	65(7) 	22(4)	26(4) 	37(5)
C(68)	41(2) 	49(2)	32(2) 	2(2)	7(2) 	23(2)
C(69)	41(6) 	53(10)	49(3) 	10(5)	-12(4) 	8(7)
C(70)	73(4) 	111(15)	122(19) 	44(18)	0(6) 	58(4)
C(71)	119(2) 	119(2)	119(2) 	24(1)	33(1) 	41(1)
C(72)	137(2) 	137(2)	137(2) 	28(1)	38(1) 	47(1)
C(73)	121(2) 	121(2)	121(2) 	24(1)	34(1) 	42(1)
C(74)	70(7) 	108(11)	71(6) 	46(8)	-2(6) 	19(7)
C(69')	41(6) 	53(10)	49(3) 	10(5)	-12(4) 	8(7)
C(70')	73(4) 	111(15)	122(19) 	44(18)	0(6) 	58(4)
C(71')	119(2) 	119(2)	119(2) 	24(1)	33(1) 	41(1)
C(72')	137(2) 	137(2)	137(2) 	28(1)	38(1) 	47(1)
C(73')	121(2) 	121(2)	121(2) 	24(1)	34(1) 	42(1)
C(74')	70(7) 	108(11)	71(6) 	46(8)	-2(6) 	19(7)
C(75)	52(2) 	55(2)	31(2) 	2(2)	4(2) 	31(2)
C(76)	25(1) 	28(2)	39(2) 	2(1)	9(1) 	8(1)
C(77)	54(2) 	34(2)	52(2) 	4(2)	2(2) 	22(2)
C(78)	74(3) 	48(3)	89(4) 	11(2)	-2(3) 	37(2)
C(79)	57(3) 	39(2)	104(4) 	-6(2)	9(3) 	24(2)
C(80)	44(2) 	40(2)	74(3) 	-19(2)	15(2) 	8(2)
C(81)	33(2) 	38(2)	47(2) 	-4(2)	9(2) 	7(2)
C(82)	24(1) 	29(2)	30(2) 	7(1)	11(1) 	9(1)
C(83)	41(3) 	44(7)	32(2) 	9(4)	3(2) 	13(5)
C(84)	49(5) 	72(7)	53(7) 	-3(5)	10(5) 	4(4)
C(85)	69(3) 	68(3)	68(3) 	5(2)	16(2) 	17(2)
C(86)	53(5) 	86(10)	43(3) 	9(8)	0(3) 	-2(8)
C(87)	41(3) 	98(10)	70(7) 	31(7)	14(4) 	22(6)
C(88)	37(2) 	67(7)	64(6) 	30(6)	11(3) 	21(6)
C(83')	41(3) 	44(7)	32(2) 	9(4)	3(2) 	13(5)
C(84')	49(5) 	72(7)	53(7) 	-3(5)	10(5) 	4(4)
C(85')	69(3) 	68(3)	68(3) 	5(2)	16(2) 	17(2)
C(86')	53(5) 	86(10)	43(3) 	9(8)	0(3) 	-2(8)
C(87')	41(3) 	98(10)	70(7) 	31(7)	14(4) 	22(6)
C(88')	37(2) 	67(7)	64(6) 	30(6)	11(3) 	21(6)
C(89)	35(2) 	66(3)	31(2) 	-3(2)	6(2) 	16(2)
C(90)	26(2) 	43(2)	43(2) 	3(2)	1(1) 	15(1)
C(91)	45(2) 	59(3)	65(3) 	18(2)	13(2) 	31(2)
C(92)	61(3) 	57(3)	91(4) 	19(3)	13(3) 	37(2)
C(93)	59(3) 	73(3)	84(4) 	0(3)	9(3) 	44(3)
C(94)	58(3) 	96(4)	67(3) 	11(3)	22(2) 	47(3)
C(95)	40(2) 	69(3)	53(2) 	14(2)	13(2) 	29(2)
C(96)	24(2) 	42(2)	32(2) 	7(1)	-2(1) 	15(1)
C(97)	24(1) 	29(2)	40(2) 	7(1)	11(1) 	10(1)
C(98)	33(2) 	41(2)	49(2) 	16(2)	15(2) 	17(2)
C(99)	45(2) 	40(2)	71(3) 	20(2)	26(2) 	24(2)
C(100)	46(2) 	35(2)	75(3) 	0(2)	26(2) 	18(2)
C(101)	52(2) 	49(2)	50(2) 	-2(2)	16(2) 	21(2)
C(102)	41(2) 	41(2)	44(2) 	8(2)	12(2) 	20(2)
C(103)	23(1) 	31(2)	38(2) 	9(1)	11(1) 	9(1)
C(104)	32(2) 	29(2)	39(2) 	8(1)	4(1) 	12(1)
C(105)	62(3) 	50(2)	39(2) 	13(2)	3(2) 	-6(2)
C(106)	86(4) 	69(3)	47(3) 	6(2)	-8(3) 	-30(3)
C(107)	58(3) 	55(3)	65(3) 	19(2)	-1(2) 	-14(2)
C(108)	52(2) 	44(2)	64(3) 	13(2)	20(2) 	4(2)
C(109)	46(2) 	37(2)	45(2) 	8(2)	13(2) 	7(2)
C(110)	24(1) 	29(2)	39(2) 	7(1)	5(1) 	13(1)
C(111)	34(2) 	53(2)	44(2) 	21(2)	5(2) 	23(2)
C(112)	42(2) 	142(5)	126(5) 	89(4)	46(3) 	41(3)
O(39)	194(4) 	189(4)	178(4) 	30(3)	46(3) 	76(3)
C(113)	143(5) 	144(5)	101(4) 	20(4)	34(4) 	42(4)
C(114)	246(7) 	247(7)	245(7) 	44(3)	75(3) 	90(4)
C(115)	189(5) 	184(5)	189(5) 	44(3)	43(3) 	77(3)
C(116)	183(6) 	167(6)	158(6) 	38(5)	27(5) 	61(5)
______________________________________________________________________________ 
 Table 5.   Hydrogen coordinates ( x 104) and isotropic  displacement parameters (Å2x 10 3)
for ic19479_sq.
________________________________________________________________________________ 
	x 	y 	z 	U(eq)
________________________________________________________________________________ 
 
H(38)	1323	3236	7825	73
H(1)	1767	8583	7238	66
H(2)	1209	9657	7141	96
H(3)	1903	10667	6731	120
H(4)	3169	10599	6441	104
H(7A)	4941	10092	7171	111
H(7B)	4817	10636	6742	111
H(7C)	5390	10030	6703	111
H(8A)	4343	9337	5798	121
H(8B)	3753	9927	5837	121
H(8C)	3256	8952	5714	121
H(9)	1127	4895	5845	68
H(10)	250	4115	5031	87
H(11)	739	4454	4308	104
H(12)	2142	5465	4417	93
H(15A)	4715	6412	5341	145
H(15B)	3930	5722	4887	145
H(15C)	4089	5594	5471	145
H(16A)	2930	7318	5043	156
H(16B)	3287	6852	4636	156
H(16C)	4010	7529	5120	156
H(17)	4869	7982	9114	45
H(18)	6110	8942	9740	55
H(19)	6374	8778	10602	62
H(20)	5324	7685	10818	52
H(23A)	2914	7238	10272	61
H(23B)	3632	7283	10804	61
H(23C)	2730	6485	10565	61
H(24A)	3602	5528	10531	67
H(24B)	4531	6281	10814	67
H(24C)	4445	5726	10280	67
H(25)	968	5377	10273	41
H(26)	1021	4841	11015	48
H(27)	1638	3790	11107	54
H(28)	2109	3259	10430	51
H(31A)	3254	3500	9355	65
H(31B)	3029	3205	9862	65
H(31C)	3520	4159	9878	65
H(32A)	802	3030	9071	71
H(32B)	1314	2528	9411	71
H(32C)	1571	2742	8894	71
H(34)	2513	1272	8201	63
H(35)	3497	523	8200	78
H(36)	4955	1125	8100	86
H(37A)	5472	2528	8065	87
H(38A)	4524	3298	8129	63
H(34')	2655	1973	8434	63
H(35')	3023	775	8427	78
H(36')	3964	553	7922	86
H(37')	4995	1673	7728	87
H(38')	4445	2814	7628	63
H(42)	4350	9385	9069	71
H(43)	5481	10543	9671	95
H(44)	6782	11284	9441	97
H(45)	7014	10878	8656	96
H(46)	5886	9802	8056	74
H(49)	5628	4499	8267	80
H(50)	6764	3886	8343	111
H(51)	7881	4254	7919	98
H(52)	7978	5319	7477	84
H(53)	6819	5911	7372	68
H(56)	6796	7478	9332	54
H(57)	8154	8377	9911	63
H(58)	8926	9611	9713	66
H(59)	8376	9917	8919	82
H(60)	7033	9007	8329	64
H(63)	3597	3562	7036	64
H(64)	4586	2816	7023	94
H(65)	5673	3141	6583	101
H(66)	5888	4262	6184	87
H(67)	4995	5085	6251	64
H(63')	3602	3638	6831	64
H(64')	4422	2743	6654	94
H(65')	5635	3245	6256	101
H(66')	5962	4595	6078	87
H(67')	4939	5307	6179	64
H(70A)	-168	7485	5991	119
H(71A)	-803	8306	5476	143
H(72A)	-432	8258	4660	165
H(73A)	965	8137	4581	145
H(74A)	1767	7628	5239	104
H(70B)	-194	7311	5841	119
H(71B)	-1018	7643	5080	143
H(72B)	71	8937	4949	165
H(73B)	1376	8646	4747	145
H(74B)	2071	7973	5338	104
H(77)	1532	8460	8133	57
H(78)	1223	9678	8273	85
H(79)	1663	10451	9108	83
H(80)	2403	10009	9803	69
H(81)	2711	8784	9663	50
H(84)	-219	3056	6376	77
H(85)	-1717	2065	6163	86
H(86)	-2916	2467	6236	84
H(87)	-2722	3805	6581	83
H(88)	-1276	4804	6766	65
H(84')	10	2942	6686	77
H(85')	-1376	1822	6297	86
H(86')	-2769	2042	6215	84
H(87')	-2774	3396	6363	83
H(88')	-1376	4460	6627	65
H(91)	-25	8073	7106	63
H(92)	-781	8975	7306	80
H(93)	-1569	8788	7930	84
H(94)	-1665	7669	8335	82
H(95)	-930	6756	8127	62
H(98)	181	7562	8954	46
H(99)	-120	8628	9410	57
H(100)	214	8838	10305	60
H(101)	872	8015	10754	60
H(102)	1187	6956	10302	49
H(105)	-1036	3281	7453	69
H(106)	-2362	2123	7230	103
H(107)	-3120	1711	7843	84
H(108)	-2465	2378	8702	67
H(109)	-1098	3489	8936	53
H(11A)	4918	5041	8933	62
H(11B)	4510	5155	9413	62
H(11C)	5212	5932	9286	62
H(11D)	6698	7682	7405	138
H(11E)	6743	7963	6868	138
H(11F)	6505	7014	6898	138
H(11G)	-1524	6201	6397	197
H(11H)	-2477	5510	6366	197
H(11I)	-2127	6362	6770	197
H(11J)	-3311	6223	5998	293
H(11K)	-2448	7055	6162	293
H(11L)	-3102	7112	5237	222
H(11M)	-3766	6164	5071	222
H(11N)	-3132	5903	4462	258
H(11O)	-2118	6444	4813	258
H(11P)	-2765	6877	4505	258
________________________________________________________________________________ 
